# Sestrins as Biomarkers of Cellular Stress and Human Disease

**DOI:** 10.3390/cells15070651

**Published:** 2026-04-06

**Authors:** Alexander Haidurov, Andrei Budanov

**Affiliations:** 1 School of Biochemistry and Immunology, Trinity Biomedical Sciences Institute, Trinity College Dublin, Pearse Street, D02 R590 Dublin, Ireland; 2 Shemyakin-Ovchinnikov Institute of Bioorganic Chemistry of the Russian Academy of Sciences, Ulitsa Miklukho-Maklaya 16/10, 117997 Moscow, Russia

**Keywords:** Sestrins, SESN1, SESN2, SESN3, biomarkers, serum, plasma, biopsy, gene expression, human disease

## Abstract

Sestrins are an evolutionarily conserved family of stress-responsive proteins that regulate cellular metabolism, redox balance, and survival. Their expression is induced by diverse cellular stresses through activation of transcription factors such as p53, NRF2, and FOXO. Through antioxidant activity and modulation of mTORC1 and mTORC2 signalling, Sestrins limit the accumulation of reactive oxygen species, regulate metabolic pathways, and promote autophagy. In this review, we analyse published studies reporting SESN1, SESN2, and SESN3 expression in human tissues, circulation, and experimental disease models. The available evidence indicates that Sestrin levels are dynamically regulated across multiple pathologies, including metabolic, ageing, cardiovascular, inflammatory, neurodegenerative, and degenerative disorders. Notably, changes in tissue Sestrin expression are often mirrored in circulation. These observations suggest that Sestrins may serve as informative biomarkers of cellular stress and disease states, and that monitoring their expression in tissues or blood could provide insight into disease progression and therapeutic response.

## 1. Introduction

The Sestrin family of stress-responsive proteins was first identified in the 1990s. There are three Sestrin genes (SESN1–3 in humans, Sesn1–3 in rodents) that encode functionally similar proteins but are regulated by distinct transcription factors. Sestrins are expressed across a wide range of human tissues, and the ancestral Sestrin protein can be traced back to invertebrate organisms, such as *Caenorhabditis elegans* [1,2]. During vertebrate evolution, this ancestral gene appears to have undergone duplication events that gave rise to the three Sestrin genes found in vertebrates, expanding the range of cellular stresses that can regulate their expression [3].

Cellular stress is a common feature of many diseases. Cells are constantly exposed to environmental, metabolic, nutrient, hypoxic, inflammatory, and DNA-damaging stresses. Chronic cellular stress contributes to the development of metabolic, cardiovascular, neurodegenerative, inflammatory, and degenerative diseases. Therefore, cells rely on stress-responsive genes to either ameliorate or adapt to these stresses. While Sestrins are ubiquitously expressed at basal levels in most tissues [4], they are strongly induced in response to several cellular stresses.

SESN1 and SESN2 are commonly upregulated in response to DNA damage through the tumour suppressor p53 [1,5]. Oxidative stress similarly induces SESN2 expression via p53 and the antioxidant transcription factor NRF2 [6,7], while SESN3 is particularly regulated by the antioxidant transcription factors FOXO1 and FOXO3 [8,9]. In addition, hypoxia induces SESN2 expression [1]. Endoplasmic reticulum (ER) stress, which frequently occurs during metabolic dysregulation, also activates SESN2 through the transcription factors ATF6, XBP1, and ATF4 [10,11,12].

Sestrins are multifunctional intracellular proteins that function primarily in the cytoplasm and help cells manage and adapt to cellular stress. Sestrins possess antioxidant activity and can directly neutralise hydrophobic reactive oxygen species (ROS) molecules [13,14]. They may also indirectly support antioxidant signalling by activating the NRF2 transcription factor [15,16] or by supporting mitochondrial recycling [17,18]. Furthermore, Sestrins regulate the activity of the mTORC1 [19] and mTORC2 [20] complexes. As a central regulator of anabolic metabolism, mTORC1 promotes protein synthesis while suppressing autophagy [21]. Consequently, Sestrin-mediated inhibition of mTORC1 reduces global protein synthesis and promotes autophagy. Under basal conditions, Sestrin-mediated modulation of mTORC1 is controlled by leucine [22,23], thereby preventing excessive inhibition of mTORC1. However, strong stress-induced upregulation of Sestrins is proposed to override this leucine-dependent control, allowing Sestrins to effectively inhibit mTORC1 during cellular stress [21]. By regulating mTORC1 and mTORC2, Sestrins suppress protein synthesis and promote autophagy, making them key regulators of the cellular stress response.

Being targets of multiple cellular stresses, Sestrins are often induced across many pathological conditions [24,25,26]. Notably, Sestrins have also been detected in circulating blood components, including serum and plasma, suggesting that their circulating levels may reflect organism-wide stress responses and therefore hold potential as accessible biomarkers of disease. An ideal biomarker should be detectable, sensitive to disease processes, and mechanistically linked to pathology. Sestrins satisfy several of these criteria, as they are rapidly induced by cellular stress, participate directly in metabolic and oxidative stress pathways, and can be detected in circulating blood components.

Our previous work has extensively examined the roles of Sestrins in cancer [27,28]. Here, we focus on their potential as biomarkers in other human diseases by reviewing studies that measure Sestrin expression in tissue and serum samples, which may reflect systemic stress responses. This review compiles studies reporting SESN1-3 expression across tissues and diseases in humans, mice, and rats, and analyses the relationship between circulating Sestrins and their tissue expression.

## 2. Patterns of Sestrin Expression Across Tissues and Conditions

Sestrins are well known to be upregulated by cellular stress; however, there is a knowledge gap between cellular findings and tissue-level regulation. In this review, we examine trends in Sestrin regulation across a variety of tissues and conditions. Our analysis is separated into Human Studies, Mouse Studies, Rat Studies, and Human Serum and Plasma studies. Table 1, Table 2, Table 3 and Table 4 present changes in Sestrin levels across different tissues and conditions, highlighting the versatility and potential involvement of Sestrins in these conditions. Throughout this review, the term “Sestrins” is used as a collective designation for the members of the Sestrin family (SESN1, SESN2, and SESN3), unless a specific protein is explicitly indicated.

### 2.1. Sestrins in Human Studies

The first cohort of studies examined comprises biopsies and primary cells obtained from human donors. Table 1 summarises studies performed across multiple tissues and clinical conditions.

A range of cross-disciplinary studies have examined Sestrin expression across diverse human disease models. In diabetes, Sestrin levels decrease in the liver and kidneys during diabetic complications [29,30,31], whereas increased expression has been reported in skeletal muscle and peripheral blood leukocytes [32,33].

Metabolic disorders also display altered Sestrin regulation. In non-alcoholic steatohepatitis (NASH), SESN3 expression is reduced in the liver [35], while obesity, defined by elevated BMI, correlates with increased SESN2 expression [34].

Cardiovascular diseases demonstrate context-dependent changes in Sestrin expression. Peripartum cardiomyopathy and heart failure are associated with decreased cardiac Sestrin levels [37]. In contrast, increased expression has been reported in cardiac tissues from patients with aortic dissection, atrial fibrillation, and calcific aortic valve disease [36,38,39]. In endothelial injury models of atherosclerosis, ox-LDL-challenged HUVECs consistently show decreased SESN1 expression [40,41,42].

Inflammatory and infectious conditions frequently induce Sestrin upregulation. Elevated expression has been reported in ulcerative colitis, cutaneous leishmaniasis, Epstein–Barr virus infection, and COVID-19 [44,45,46,47,48]. However, reduced expression has been observed in septic intestinal dysfunction and systemic lupus erythematosus [43,49].

Ageing is generally associated with reduced Sestrin expression across multiple tissues, including knee cartilage, skeletal muscle, HUVECs, and serum [50,51,52,53]. However, Sestrins are upregulated in differentiated and senescent T cells [55,56], and increased expression has also been observed in peripheral blood cells following progesterone treatment or X-ray exposure [54,56].

Neurological diseases similarly display altered Sestrin regulation. Increased expression has been reported in the brain in temporal lobe epilepsy, Parkinson’s disease, and amyotrophic lateral sclerosis [57,58,60]. However, one study reported no significant change in SESN3 expression in Parkinson’s disease [59].

Respiratory diseases also demonstrate elevated Sestrin levels. Increased expression has been reported in lung tissue from patients with chronic obstructive pulmonary disease and in sputum samples from patients with asthma [61,63]. In contrast, SESN2 expression in nasal polyposis was reported to decrease, although this change was not statistically significant [62].

Several degenerative tissue conditions are associated with reduced Sestrin expression. Decreased levels have been reported in cataracts, osteoarthritis, ligamentum flavum hypertrophy, liver fibrosis, and intervertebral disc degeneration [50,64,65,67,68,69,70]. Conversely, increased expression has been observed in gastric intestinal metaplasia and kyphoscoliotic Ehlers–Danlos syndrome [66,71].

Environmental stress can also modulate Sestrin expression. Ultraviolet B (UVB) exposure increases SESN2 levels in skin explants [72].

Physiological interventions further influence Sestrin regulation. Acute resistance exercise and resistance training increase Sestrin expression in skeletal muscle [73], and ultra-endurance cycling athletes display elevated Sestrin expression in mesenchymal stem cells following prolonged training [83]. In contrast, dietary protein supplementation does not significantly alter Sestrin expression [75,76], while muscle disuse results in reduced SESN2 levels following 14 days of immobilisation [74].

Various experimental treatments also induce Sestrin expression. Angiotensin II stimulation of endothelial progenitor cells and zinc oxide-induced oxidative stress in BJ fibroblasts both increase Sestrin levels [77,78].

Radiation exposure represents another potent regulator of Sestrin expression. Radiotherapy increases Sestrin levels in peripheral blood leukocytes [81,82]. However, Sestrin expression in PBMCs can dynamically shift from upregulation to downregulation depending on the time elapsed after radiotherapy [79,84].

Collectively, these findings demonstrate that Sestrin expression is dynamically regulated across human diseases and physiological conditions. In general, Sestrin levels tend to decrease in chronic pathological conditions, including type 2 diabetes complications, ageing, and tissue degeneration. In contrast, Sestrin expression is frequently increased in neurological and respiratory diseases, as well as during inflammatory, hypoxic, and epithelial-stress conditions, such as cardiovascular injury, inflammatory diseases, and UVB exposure. Physiological interventions such as exercise and endurance training also induce Sestrin expression, whereas dietary supplements do not appear to alter Sestrin levels.

Together, these observations support the concept that Sestrins function as stress-responsive protective proteins. Reduced Sestrin expression in chronically damaged tissues may therefore contribute to impaired cellular stress adaptation and tissue degeneration.

### 2.2. Sestrins in Mouse Studies

The second cohort of studies examined comprises tissue samples and primary cells obtained from mouse models. Table 2 summarises studies performed across multiple tissues, disease models, and experimental conditions.

Mouse studies constitute the largest body of in vivo literature on Sestrins, with Sesn2 emerging as the most frequently examined family member. Across these models, their regulation is strongly context- and tissue-dependent, although several broad trends are apparent.

In diabetic models, Sestrins are predominantly downregulated. Reduced Sesn2 expression has been reported in the heart, kidney, and liver across multiple mouse models of diabetes [85,87,88,89,90]. However, one study of streptozotocin-induced diabetes reported increased cardiac Sesn2 expression [86], indicating that regulation may vary according to disease stage, model, or tissue context.

Metabolic disorders also demonstrate heterogeneous Sestrin regulation. High-fat diet- and Western diet-associated models generally show reduced expression in the brain, heart, and liver [34,91,94,96,97,110], and alcohol-induced hepatic steatosis similarly decreases hepatic Sestrins [98]. However, shorter-term high-fat feeding was associated with increased hepatic Sesn2 expression at 8 weeks [93]. In skeletal muscle, high-fat diet models show reduced Sesn3 but increased Sesn2 expression [100,101,102]. By contrast, maternal obesity increased placental Sesn2 expression [99]. Experimental metabolic stress also induced Sesn2, including glucose starvation in embryonic fibroblasts [103] and endoplasmic reticulum storage stress in PiZ hepatocytes [71].

Cardiovascular models frequently show increased Sestrin expression. Upregulation has been reported in atherosclerotic aorta [104], in the prefrontal cortex following myocardial infarction [105], and in the heart and lung in heart failure with preserved ejection fraction [106]. Similarly, pressure overload, diabetic cardiomyopathy, and doxorubicin-induced cardiotoxicity increase cardiac Sestrin expression [107,108,109]. Increased levels have also been observed in macrophages and monocytes derived from atherosclerotic aorta and infarcted myocardium [111,175].

Inflammatory and infectious models also tend to induce Sestrins. Cecal ligation and puncture- or lipopolysaccharide-based sepsis models increase expression in the brain, heart, alveolar macrophages, and splenic dendritic cells [112,113,114,116,118,121,122]. Dextran sulphate sodium-induced colitis similarly increases Sestrin expression in colonic immune cells [119], and trained immunity induced by BSNP treatment increases Sesn1 expression [120]. However, studies associated sepsis-induced liver injury with reduced hepatic Sesn2 [117] and experimental autoimmune encephalomyelitis with decreased colonic Sesn3 [115].

Ischemic and injury models mostly show increased Sestrin expression, with some notable exceptions. Ischemia–reperfusion injury generally increases expression in the brain, heart, and liver [134,135,136,137,141,143]. Similarly, noise-induced hearing loss increased cochlear Sesn2 [138], spared nerve injury increased Sesn2 in lumbar dorsal root ganglia and sciatic nerve [151], and cholestatic liver injury increased hepatic Sestrins [146,147]. In contrast, acute kidney injury and corneal epithelial wound healing were associated with reduced Sesn2 expression [140,142]. Acetaminophen-induced liver injury showed conflicting results, with one study reporting increased and another decreased Sesn2 expression [144,145]. Pulmonary fibrosis models show increased Sesn3 but reduced Sesn2 expression in the lung [148,149,150]. Skin injury increased Sesn2 expression in burn wounds [153].

Ageing broadly correlates with reduced Sestrin expression in mice. Decreased expression has been reported in the heart, nucleus pulposus, prostate, and skeletal muscle of aged mice [123,124,125,126,127,128,130,132]. Accelerated ageing in Foxo1/3/4 knockout mice also reduced Sesn3 expression in the brain [157]. However, some studies report increased expression with ageing. One sarcopenia study reported increased Sesn2 in gastrocnemius muscle [129], and exercise training in aged mice increased skeletal muscle Sesn2 expression [130]. In addition, splenic CD4+ T cells from aged mice showed increased expression of all three Sestrins [56].

Neurodegenerative models show variable Sestrin expression. In Alzheimer’s disease models, studies have reported both decreased [154] and increased [155] cortical Sesn2 expression, whereas one study found increased Sesn1 expression [156]. Degenerative disease models outside the nervous system show increased expression. Duchenne muscular dystrophy and osteogenesis imperfecta are both associated with increased Sesn2 in affected tissues [71,158].

Musculoskeletal remodelling and atrophy models demonstrate conflicting changes in Sestrin expression. Immobilisation reduced Sestrins in gastrocnemius, soleus, and tibialis anterior muscles [127,159,164]. However, other disuse and denervation studies reported increased Sesn2 in the gastrocnemius muscle, particularly at earlier time points [160,161,162]. Microgravity-induced muscle adaptation during 30-day spaceflight increased Sesn1 expression [163], supporting the concept that Sestrins respond dynamically to muscle stress and remodelling conditions.

Several physiological and experimental interventions in mice alter Sestrin expression. Acute restraint stress increased Sesn1 expression in the lung [168], whereas chronic restraint stress reduced Sestrins in skeletal muscle [169]. Exercise increased Sestrin expression in brown adipose tissue, subcutaneous white adipose tissue, and quadriceps muscle [92,171,172], while 24-h fasting induced Sesn1 in quadriceps [173]. By contrast, alcohol-induced intestinal injury and acute ethanol exposure in mice reduced Sesn2 expression in the colon and skeletal muscle, respectively [139,170]. Ultraviolet exposure, cigarette smoke extract, and tunicamycin-induced endoplasmic reticulum stress increased Sesn2 expression in the studied tissues [165,167,174].

Consistent with human studies, Sestrins are frequently downregulated in diabetic mouse models. In cardiovascular disease, Sestrin regulation varies between studies. Although increased Sestrin expression has been reported in some human cardiovascular pathologies, a greater number of mouse studies report upregulation of Sestrins in cardiovascular injury and disease models. Inflammatory conditions likewise commonly increase Sestrin expression in mice, similar to findings in human studies. Ageing is broadly associated with reduced Sestrin expression in both humans and mice, whereas neurological disease models in both species more often show increased Sestrin levels. Degenerative disorders show different patterns between human and mouse studies. Although reduced Sestrin expression, particularly in osteoarthritis, has been more consistently reported in human tissues, mouse models of degenerative disease more often show increased Sestrin levels. Exercise also increases Sestrin expression in both humans and mice. Overall, these observations suggest that Sestrins are often reduced in chronic diseases but increased during acute or rapidly evolving tissue stress.

### 2.3. Sestrins in Rat Studies

The third cohort of studies examined comprises tissue samples and primary cells obtained from rat models. Table 3 summarises studies performed across multiple tissues, disease models, and experimental conditions.

Of the three cohorts, rat studies indicate that Sestrin regulation is tissue- and time-dependent.

In diabetic models, Sestrins are generally decreased. Reduced Sesn2 expression has been reported in the kidney, retina, heart, and cardiac fibroblasts in streptozotocin-induced diabetes and related diabetic complications [178,179,180,181,182,183,184]. Consistent with this, myocardial ischemia–reperfusion combined with streptozotocin-induced diabetes was associated with reduced cardiac Sesn2 [177]. However, Zucker diabetic fatty rats showed increased cardiac Sesn2 expression [176]. Diet-induced obesity also reduced Sesn2 in the aorta and heart [184].

Sestrin regulation varies among rat cardiovascular models. In doxorubicin-induced cardiomyopathy, cardiac Sesn2 protein was decreased, whereas Sesn1 mRNA was increased [186]. Following acute myocardial infarction, Sesn2 was increased in the heart from 1 to 14 days after infarction but returned to control levels by day 28 [187]. Cardiac arrest also increased Sesn2 in the hippocampus immediately after injury [185]. Angiotensin II treatment, used as a model of hypertensive vascular stress, reduced Sesn2 in endothelial progenitor cells [188] and decreased Sesn1 in cardiac fibroblasts [218]. Similarly, phenylephrine-induced cardiomyocyte hypertrophy reduced Sesn2 [189].

Inflammatory models similarly show variable expression. In the heart, lipopolysaccharide treatment increased Sesn2 at 6 h [190], whereas a 24-h model showed reduced Sesn2 [191]. In a respiratory inflammatory model, ovalbumen-induced asthma increased Sesn2 in airway tissues [208].

Ageing and exercise studies in skeletal muscle also indicate tissue-specific regulation. Lifelong aerobic training increased Sesn1, Sesn2, and Sesn3 in quadriceps, but no significant changes were detected in the soleus muscle [214]. Aerobic exercise training did not alter Sesn2 in gastrocnemius [215].

Ischemic and injury models show tissue-dependent regulation. In the brain, cerebral ischemia–reperfusion and related stroke models consistently increased Sesn2 in the cerebral cortex, hippocampus, and ischemic cortical penumbra [192,193,194,195,196]. In contrast, myocardial ischemia–reperfusion reduced Sesn1 in the heart [197]. Renal ischemia–reperfusion studies were inconsistent, with two reporting decreased Sesn2 [198,199] and one reporting increased Sesn2 [200].

Additional injury models also showed variable responses. Adriamycin-induced nephropathy did not alter Sesn2 expression at early time points but was associated with reduced renal Sesn2 at later stages [201], whereas paraquat-induced acute kidney injury increased Sesn2 [202]. Sodium arsenite-induced liver injury increased hepatic Sesn2 [203], while bleomycin-induced pulmonary fibrosis reduced Sesn2 in the lung [204].

Neurological disease models more commonly showed increased expression. Sevoflurane-induced cognitive dysfunction increased Sesn1 in the hippocampus [205]. Increased Sesn2 was also reported in malonic acid-induced Huntington’s disease [206], amyloid-β-treated cortical neurons [207], and cortical neurons following bicuculline and 4-aminopyridine stimulation [217].

By contrast, degenerative and musculoskeletal remodelling models did not show consistent induction. Sesn2 was unchanged in the spinal cord in an osteoarthritis pain model [209]. Skeletal muscle hypertrophy and hindlimb suspension were associated with reduced expression [210,211]. Early-life stress reduced Sesn3 in skeletal muscle, whereas chronic unpredictable stress had no significant effect [216]. In other physiological interventions, short-term calorie restriction did not alter hepatic Sestrin expression [212], whereas triiodothyronine increased hepatic Sesn2 [213].

Overall, rat studies indicate that Sestrin regulation is highly dependent on tissue, stimulus, and time point. Nevertheless, several broad patterns emerge. Diabetic complications are generally associated with reduced Sestrin expression, consistent with observations in human and mouse studies. Cerebral ischemia–reperfusion frequently induces Sesn2 in rats, paralleling findings in mice. Neurological disease models across human, mouse, and rat studies also commonly report increased Sestrin expression. Bleomycin-induced pulmonary fibrosis is also associated with reduced Sesn2 in both rodents. Inflammatory responses appear to be time-dependent, with early induction followed by later suppression in some models. Across all three cohorts, exercise is frequently associated with increased Sestrin expression, although this response is not universal. Broadly, these findings support the concept that Sestrins function as stress-responsive regulators whose expression is often reduced in chronic pathological conditions but induced during acute injury, in a tissue- and time-dependent manner.

### 2.4. Circulating Sestrins in Human Serum and Plasma

The fourth cohort of studies examined comprises circulating Sestrins measured in human serum and plasma samples. Table 4 summarises studies reporting circulating Sestrin concentrations across multiple clinical conditions. An extended version of Table 4, including country of origin, assay kit details, age, and gender ratio, is provided in Supplementary Table S1.

Circulating Sestrins measured in serum or plasma may have clinical relevance, as these specimens are readily accessible and suitable for repeated sampling. This allows circulating levels to be evaluated as potential biomarkers for disease detection and monitoring.

Direct comparisons of circulating Sestrin studies are challenging due to substantial variability in reported concentrations, which range from approximately 1 to 20 ng/mL. This variability likely reflects differences in analytical methods and assay kits used across studies. The studies presented in Table 4 employed a range of more than 15 distinct ELISA kits. In addition, these studies were conducted across multiple research disciplines, countries, and age groups. SESN2 is the most frequently reported circulating Sestrin, possibly reflecting its wider availability in commercial detection kits. Despite these differences between studies, several recurring patterns are observed when comparing test groups with controls.

In diabetes and metabolic disease, circulating SESN2 is most often decreased. Reduced serum or plasma SESN2 has been reported in type 2 diabetes, diabetic nephropathy, dyslipidaemia, obesity, and paediatric obesity [220,221,222,223,224,227,228]. However, one study of diabetic neuropathy, a diabetes complication involving damage to peripheral nerves, reported increased serum SESN2 compared with controls [219].

Cardiovascular disorders frequently show increased circulating levels. Plasma SESN2 is elevated in aortic dissection, advanced carotid atherosclerosis, coronary artery disease, and congenital heart disease with or without heart failure [36,229,230,232]. Plasma SESN1 and SESN3 are also increased in stable angina, unstable angina, and acute myocardial infarction [231]. An exception is type 2 diabetes with coronary heart disease, where plasma SESN2 was reduced [226], suggesting that chronic metabolic disease may override the increase in circulating levels otherwise seen in cardiovascular stress.

Inflammatory and infectious diseases show variable circulating levels. Increased serum SESN2 has been reported in Kawasaki disease, sepsis, and septic shock with or without septic cardiomyopathy [234,236,238]. In contrast, reduced circulating levels have been reported in Hashimoto’s disease, rheumatoid arthritis, and one septic shock cohort [233,235,237].

Neurological and respiratory conditions show some parallels between tissue and circulating studies. Serum SESN2 is elevated in Alzheimer’s disease, mild cognitive impairment, and Parkinson’s disease [243,245]. In contrast, reduced circulating SESN2 has been reported in major depressive disorder and relapsing-remitting multiple sclerosis [244,246]. In respiratory disease, circulating SESN2 levels are elevated in asthma and COPD [247,248,249], consistent with increased expression in lung tissue and sputum in COPD and severe asthma.

Ageing, frailty, and musculoskeletal degeneration show less consistent patterns between tissue and circulating measurements. Ageing tissues commonly exhibit reduced Sestrin expression, and decreased circulating levels of SESN1 and SESN2 have been reported in Rockwood frailty and sarcopenia cohorts [242,250]. However, one study using the Fried frailty phenotype reported increased circulating SESN1 [241].

Reproductive, sleep-related, and physiological conditions also show altered circulating levels. Serum SESN2 levels are elevated in endometrial polyps, ovarian endometrioma, severe preeclampsia, and uterine leiomyoma [252,253,259,261]. Reduced SESN2 has been reported in threatened preterm labour and in two cohorts with polycystic ovary syndrome [255,256,260], although two additional PCOS studies reported increased circulating SESN2 [257,258]. Resistance training increases serum SESN2 [262], consistent with increased Sestrin expression reported in tissue studies following exercise. Sleep disorders show distinct patterns, with reduced SESN2 in chronic insomnia [263] and increased circulating SESN2 in obstructive sleep apnea [264,265], reinforcing the trend that these proteins decrease during chronic pathology but upregulate during intermittent stress.

Several patterns observed in tissue studies are also seen in circulating Sestrin measurements. Diabetes and diabetic complications frequently show reduced expression in both tissues and circulation. Cardiovascular conditions such as aortic dissection and coronary artery disease show increased levels in tissue and increased circulating levels. Respiratory diseases, including COPD and asthma, are associated with increased levels in lung tissue, sputum, and the circulation. Ageing tissues frequently show reduced Sestrins, and lower circulating SESN2 has been reported in frailty and sarcopenia cohorts. Exercise increases Sestrin expression in skeletal muscle and circulating SESN2 following resistance training.

While some trends are inconsistent across studies, differences in tissues analysed, assay methods, time points, and study populations likely contribute to this variability. While not all findings mirror these trends exactly, recurring parallels are observed, suggesting that Sestrins may serve as accessible systemic indicators of the same stress-responsive pathways identified in biopsy and experimental studies.

## 3. Discussion

### 3.1. Patterns of Sestrin Regulation Across Species and Tissues

Across the compiled human, mouse, and rat studies, Sestrin expression is significantly altered across diseases, but the direction and magnitude of change depend on disease type, stage, and tissue context. Table 5 summarises these patterns, highlighting both consistent and variable trends across conditions and model systems.

Overall, Sestrin levels tend to decrease in chronic disease states, whereas they often increase in response to acute or stress-related stimuli. Across all three species, diabetes, particularly with complications, is predominantly associated with decreased Sestrin expression. Ageing is consistently associated with decreased Sestrin expression across multiple tissues, except in immune cells, where Sestrin levels are increased, likely reflecting heightened metabolic and oxidative stress. Likewise, Sestrins tend to be lower in degenerative contexts in humans, consistent with impaired stress-response capacity. Cardiovascular and ischemic injury models, especially in rodents, demonstrate increased Sestrin expression, likely reflecting activation of protective responses to oxidative and hypoxic stimuli. Exercise also consistently induces Sestrin expression across human and mouse studies. Respiratory conditions likewise more often show increased Sestrin expression. Overall, circulating Sestrin levels frequently parallel tissue-level changes and, in several cohorts, correlate with disease severity, supporting their potential utility as biomarkers of systemic stress.

In contrast, several disease categories exhibit substantial variability in Sestrin regulation. Cardiovascular and inflammatory conditions show mixed patterns of up- and downregulation in tissue studies, likely reflecting differences in tissue type, disease stage, and experimental model. Similarly, ischemia and injury models demonstrate mixed responses, with Sestrin expression varying depending on the timing and severity of injury. Neurological conditions more frequently show increased Sestrin expression, although findings are not entirely consistent across models. These inconsistencies likely arise from tissue-specific effects, temporal dynamics, and methodological variability between studies.

Taken together, the evidence supports the view that Sestrins are stress-inducible proteins that are upregulated during acute stress but may become depleted, dysregulated, or insufficient in chronic disease states, thereby reflecting the state of cellular and systemic stress.

### 3.2. Sources of Inconsistency in Reported Sestrin Expression

Differences in experimental conditions and sample quality complicate Sestrin expression analysis and contribute to apparently contradictory findings. As our analysis spans studies across many tissues and diseases, these discrepancies can largely be explained by several key factors, including disease stage and severity, experimental models, tissue- and cell-type-specificity, and methodological variability.

Disease stage is a major determinant of Sestrin expression. Generally, conditions are observed at different stages of the response. For example, radiation exposure in human patients initially upregulates SESN1 in peripheral blood cells [79,81,82]. In contrast, prolonged exposure correlates with significantly lower SESN1 [79,84]. Similarly, in acute myocardial infarction models, Sesn2 is increased in the early post-injury phase but returns to baseline at later time points [187]. Comparable time-dependent patterns are observed in cardiac inflammation and liver injury models, where early upregulation is followed by suppression as injury progresses [144,145,190,191]. High-fat diet studies suggest a time-dependent effect on Sestrin regulation, whereby short-term exposure (3–8 weeks) is associated with increased Sesn2 expression [92,93], while longer-term HFD models (≥12 weeks) more consistently report reduced Sestrin levels in metabolically compromised tissues [34,94,100].

Disease severity also influences Sestrin levels. In human studies, circulating Sestrin expression may remain unchanged in early disease but become significantly altered with increasing severity. For example, SESN2 levels are elevated only in advanced carotid atherosclerosis [229] and are higher in COPD with emphysema compared to COPD alone [248]. Meanwhile, progressively reduced SESN2 levels are associated with worsening diabetic nephropathy [220,221,222].

Differences in experimental models further contribute to variability. In rats, all studies showed a decrease in Sesn2 across all diabetic conditions and tissues, except the Zucker diabetic fatty rat model, which showed an increase in Sesn2 mRNA [176]. Alzheimer’s disease models show conflicting results, with decreased Sesn2 expression at 9 months [154] but increased expression at 12 months in APPswe/PSEN1dE9 mice [155]. As amyloid plaque deposition begins at approximately 6 months in this model [266], these discrepancies may reflect differences in disease stage, age, or tissue sampling.

Tissue- and cell-type specificity also play an important role. Opposing expression patterns can be observed between organs subjected to the same stressor. For example, ischemia–reperfusion injury increases Sesn2 in the brain [192,193,194,195,196], but decreases Sestrins in the heart and kidney [197,198,199]. Ageing is associated with reduced Sestrin expression in tissues such as skeletal muscle, heart, and prostate [52,123,126], whereas CD4+ and CD8+ T cells show increased Sestrin expression with age [55,56].

Methodological variability represents an additional source of inconsistency. Differences between mRNA and protein measurements, as well as antibody specificity, may lead to divergent results. For example, in human diabetes, protein assays show decreased SESN2 [29,30], whereas qPCR indicates increased SESN1 and SESN3 expression [32,33]. Similarly, discrepancies in Sesn2 detection in kidney injury may reflect antibody differences, with two studies using the same antibody reporting decreased Sesn2 [198,199], and another using a different antibody reporting an increase [200]. In addition, the studies summarised in this review span multiple research disciplines and methodological approaches, including diverse patient cohorts from different geographical and ethnic populations, further contributing to heterogeneity in the reported findings.

Collectively, these discrepancies can be attributed to differences in disease stage and severity, experimental models, tissue- and cell-type-specificity, and methodological variability, highlighting the need for standardised approaches and careful interpretation of Sestrin expression data.

### 3.3. Limitations and Knowledge Gaps

The current literature is limited by predominantly cross-sectional studies, small sample sizes, and insufficient methodological standardisation. In addition, key knowledge gaps include the lack of longitudinal studies and a limited understanding of disease-stage-specific regulation.

The studies displayed in this review are predominantly cross-sectional and generally have small sample sizes. In studies with higher sample sizes, such as the circulatory Sestrins, we have learned that the severity of disease corresponds to the magnitude of Sestrin change. Longitudinal studies with repeated measurements may further clarify the relationship between systemic stress and circulating Sestrin levels. For example, a longitudinal study of aneurysmal subarachnoid haemorrhage reported elevated circulating SESN2 on admission, peaking two days after admission and declining by day seven [267]. Therefore, while Sestrins are potential stress markers that change with disease progression, their clinical use as biomarkers remains challenging, as the expression patterns and dynamics that reliably inform prognosis and diagnosis are not yet well defined.

The underrepresentation of certain diseases in current research must be addressed through further studies. Based on the evidence collected, inflammatory and autoimmune diseases show variable Sestrin expression patterns. This variability is not unexpected, as different conditions are likely to elicit distinct biological responses. However, additional research focusing on diseases such as multiple sclerosis, rheumatoid arthritis, and other chronic inflammatory syndromes is needed to better characterise Sestrin expression in these conditions. Such studies would help determine whether Sestrins can be reliably utilised as biomarkers within this group of diseases.

Another important consideration is that most studies report group-level differences rather than uniform changes across individuals. Observed alterations in Sestrin levels therefore represent statistical trends within cohorts, and individuals within the same cohort may show substantially different Sestrin concentrations.

Although Sestrins are broadly induced or suppressed across many conditions, a limitation of Sestrins as diagnostic biomarkers is their lack of disease specificity. As stress-responsive proteins induced by diverse stimuli, including oxidative stress, hypoxia, and metabolic dysregulation, our evidence indicates that Sestrin expression is significantly altered across a wide range of pathological conditions. However, changes in Sestrin levels may reflect a general stress response rather than a disease-specific signature. While this limits their utility as standalone diagnostic markers, Sestrins may still provide valuable information regarding disease severity, progression, or treatment response, particularly when used in combination with more disease-specific biomarkers.

Future studies using standardised assays and protocols may help establish Sestrins as clinical indicators of cellular stress. Investigation of additional biological fluids may also be informative. For example, although SESN2 levels are elevated in the circulation of patients with obstructive sleep apnea, a separate study also reported increased SESN2 levels in the urine of OSA patients [268].

### 3.4. Therapeutic Implications and Biomarker Potential

Sestrins play a central role in regulating cellular stress responses, including mTOR signalling and oxidative stress pathways, making them relevant targets for therapeutic modulation.

The current focus of Sestrin-related drug development is on leucine mimetics. Rather than modulating Sestrin expression, these directly counteract Sestrin’s mTOR-inhibitory activity and show promise for alleviating pathological mTOR suppression [269,270]. Furthermore, protein-rich supplementation, although expected to modulate Sestrin activity through increased intracellular leucine availability, does not appear to alter Sestrin expression in the studies included in this review [75,76]. Therefore, the potential of Sestrin as a biomarker may be underappreciated. Current therapies primarily target Sestrin function (for example, GATOR2 interaction), but these functional changes are difficult to measure in clinical settings, whereas Sestrin expression is readily quantifiable.

Sestrin expression represents a measurable output that may be used to assess therapeutic efficacy. For example, metformin, the widely used anti-diabetic drug, has been confirmed to upregulate SESN2 [271]. As our evidence shows, Sestrin levels decline in diabetes and related complications. Therefore, metformin-induced Sestrin expression may serve as an indicator of treatment response. GLP-1 receptor agonists are gaining widespread clinical use, including semaglutide (Ozempic). Another GLP-1 agonist, liraglutide, has been shown to increase Sesn2 expression in mouse liver [96] and rat skeletal muscle cells [272]. Investigating Sestrin expression in response to such emerging therapies may help clarify its utility as a biomarker of therapeutic efficacy.

Exercise is among the most effective interventions for metabolic pathologies and age-related mobility decline, and Sestrins have been proposed as essential molecular mediators of exercise benefits [273]. In the evidence we describe in this review, we frequently observe reduced Sestrin levels in diabetic, metabolic, and age-related pathologies. Furthermore, in this review, we observe that Sestrins are upregulated in response to exercise across multiple tissues and in circulation [73,83,130,171,172,175,214,262]. Therefore, Sestrins may serve as markers of exercise efficacy.

Beyond their role as biomarkers, Sestrins play an important role in counteracting pathology. Sestrin expression has been linked to prognosis in cancer [274,275,276]. From the studies we examined in this review, SESN2 decreased with the severity of diabetic nephropathy [220], low serum SESN2 levels were related to the increased risk of T2DM-CHD [226], and circulatory Sestrin levels were predictive of CAD severity [231]. Therefore, Sestrins may serve not only as biomarkers but also as functionally relevant regulators of disease progression and as potential therapeutic targets.

Accordingly, targeting Sestrins may offer a means to modulate key protective processes such as autophagy, redox balance, and cell survival. Meanwhile, their expression may serve as a measurable indicator of therapeutic response.

## 4. Conclusions

Sestrins are stress-inducible proteins that are consistently upregulated during acute stress but reduced or dysregulated in chronic conditions such as diabetes, ageing, and degenerative diseases. Across studies, their expression is strongly context-dependent, varying with disease stage, severity, and tissue type. Despite some variability, key trends emerge: Sestrins decline in metabolic and age-related disorders, increase in response to exercise, and circulating Sestrin levels often mirror tissue-level changes. While their lack of disease specificity limits their diagnostic use, Sestrin expression represents a measurable marker of cellular stress with potential to reflect disease severity and therapeutic response, particularly when combined with other disease-specific biomarkers.

## Figures and Tables

**Table 1:** Human studies reporting Sestrin expression changes. Summary of published studies reporting SESN1, SESN2, or SESN3 expression changes measured in human tissue biopsies or primary cells across various clinical conditions. “Test (*n*) vs. Comparator (*n*)” indicates the groups used for Sestrin comparison. “Sestrin change” denotes the direction of expression relative to the study-defined control group. Symbols indicate the reported direction of change (↑, upregulation; ↓, downregulation; –, no change). Statistical significance is indicated by * where *p* < 0.05 (NS, not significant; NR, not reported). “Assay” denotes the method used to measure Sestrin expression. Abbreviations: AF, atrial fibrillation; ALS, amyotrophic lateral sclerosis; Ang II, angiotensin II; AoD, aortic dissection; BMI, body mass index; CAVD, calcific aortic valve disease; CD, cluster of differentiation; COPD, chronic obstructive pulmonary disease; COVID-19, coronavirus disease 2019; DKD, diabetic kidney disease; DN, diabetic nephropathy; DPN, diabetic peripheral neuropathy; ELISA, enzyme-linked immunosorbent assay; EBV, Epstein–Barr virus; EPCs, endothelial progenitor cells; EV, extracellular vesicles; FC, flow cytometry; GIM, gastric intestinal metaplasia; GOLD, Global Initiative for Chronic Obstructive Lung Disease; HF, heart failure; HUVEC, human umbilical vein endothelial cells; IDD, intervertebral disc degeneration; IF, immunofluorescence; IHC, immunohistochemistry; kEDS, kyphoscoliotic Ehlers–Danlos syndrome; LC-EV, long COVID-derived extracellular vesicles; LCL, localized cutaneous leishmaniasis; LFH, ligamentum flavum hypertrophy; MA, microarray analysis; MPC, milk protein concentrate; NAFLD, non-alcoholic fatty liver disease; NASH, non-alcoholic steatohepatitis; NP, nasal polyposis; NuP, nucleus pulposus; ox-LDL, oxidised low-density lipoprotein; PA, proliferated-arrested EBV-infected B cells; PBMC, peripheral blood mononuclear cells; PD, Parkinson’s disease; PPCM, peripartum cardiomyopathy; PP, proliferated-proliferated EBV-infected B cells; RDA, recommended dietary allowance; RE, resistance exercise; RET, resistance training; RNA-seq, RNA sequencing; RT, radiotherapy; RT-qPCR, reverse transcription quantitative polymerase chain reaction; scRNA-seq, single-cell RNA sequencing; SLE, systemic lupus erythematosus; T2DM, type 2 diabetes mellitus; TLE, temporal lobe epilepsy; UVB, ultraviolet B; WB, Western blot; ZnO, zinc oxide.

Tissue	Condition	Test (*n*) vs. Comparator (*n*)	Sestrin Change	Assay	Ref.
**Diabetes**					
Kidney (glomeruli)	Diabetic nephropathy (T2DM complication)	DN (*n* = 15) vs. control (*n* = 15)	SESN2 ↓ *	IHC	[29]
Kidney (glomeruli and tubules)	Diabetic kidney disease	T2DM with DKD (*n* = 11) vs. control (*n* = 3)	SESN2 ↓ *	IHC	[30]
Liver	Type 2 diabetes	NAFLD + T2DM (*n* = 6) vs. control (*n* = 8)	SESN1 ↓ *	MA	[31]
Skeletal muscle (vastus lateralis)	Type 2 diabetes	T2DM (*n* = 10) vs. control (*n* = 12)	SESN3 ↑ *	RT-qPCR	[32]
Peripheral blood leukocytes	Diabetic neuropathy (T2DM complication)	DPN (*n* = 20) vs. diabetes without DPN (*n* = 20)	SESN1 ↑ * SESN3 ↑ *	RT-qPCR	[33]
**Metabolic**					
Heart (left ventricle)	Obesity	Obese BMI (*n* = 9) vs. ideal BMI control (*n* = 3)	SESN2 ↑ (NR)	WB	[34]
Liver	Nonalcoholic steatohepatitis (NASH)	NASH (*n* = 3) vs. control (*n* = 3)	SESN3 ↓ *	WB	[35]
Liver	Nonalcoholic steatohepatitis (NASH)	Fibrosis stages F1, F2, F3, F4 vs. F0 (*n* = 3/group)	SESN3 ↓ *	IHC	[35]
**Cardiovascular**					
Aorta	Aortic dissection	AoD patients (*n* = 12) vs. control (*n* = 9)	SESN2 ↑ *	WB	[36]
Heart	Peripartum cardiomyopathy	PPCM (*n* = 4) vs. control (*n* = 5)	SESN2 ↓ *	WB	[37]
Heart	Heart failure	HF (*n* = 6) vs. control (*n* = 5)	SESN2 ↓ *	WB	[37]
Heart (atrial appendages)	Atrial Fibrillation	Permanent atrial fibrillation (*n* = 19) vs. sinus rhythm (*n* = 23)	SESN1 ↑ * SESN2 ↑ * SESN3 ↑ *	WB; IF	[38]
Heart (aortic valve tissue)	Calcific aortic valve disease	CAVD (*n* = 20) vs. normal aortic valves (*n* = 10)	SESN2 ↑ *	RT-qPCR; WB; IF	[39]
HUVEC	Atherosclerosis model (ox-LDL endothelial injury)	Ox-LDL-treated (*n* = 3) vs. control (*n* = 3)	SESN1 ↓ *	RT-qPCR; WB	[40]
HUVEC	Atherosclerosis model (ox-LDL endothelial injury)	Ox-LDL-treated (*n* = 3) vs. control (*n* = 3)	SESN1 ↓ *	RT-qPCR; WB	[41]
HUVEC	Atherosclerosis model (ox-LDL endothelial injury)	Ox-LDL-treated (*n* = 3) vs. control (*n* = 3)	SESN1 ↓ *	WB	[42]
**Inflammatory/Infectious**					
Blood	Septic intestinal dysfunction	Septic intestinal dysfunction patients (*n* = 6) vs. control (*n* = 6)	SESN2 ↓ *	RT-qPCR	[43]
Colon	Ulcerative colitis	Inflamed colon (*n* = 10) vs. non-inflamed control (*n* = 10)	SESN2 ↑ *	RT-qPCR	[44]
Skin	Cutaneous leishmaniasis	LCL (*n* = 21) vs. control (*n* = 7)	SESN2 ↑ *	RNA-seq	[45]
Aortic smooth muscle cells	Extracellular vesicles isolated from Long COVID patients	LC-EV-treated cells (*n* = 3) vs. control (*n* = 3)	SESN1 ↑ *	WB	[46]
HUVEC	Extracellular vesicles isolated from Long COVID patients	LC-EV-treated cells (*n* = 3) vs. control (*n* = 3)	SESN2 ↑ *	WB	[46]
B cells (CD19+; PBMC-derived)	Epstein–Barr virus infection (B-cell immortalization model)	PA cells (*n* = 3) vs. PP cells (*n* = 3)	SESN1 ↑ *	RT-qPCR; WB	[47]
PBMC	COVID-19	COVID-19 patients (*n* = 5) vs. control (*n* = 5)	SESN1 ↑ *	RT-qPCR	[48]
PBMC	Systemic lupus erythematosus	SLE patients (RT-qPCR *n* = 5; WB *n* = 3) vs. control (RT-qPCR *n* = 5; WB *n* = 3)	SESN1 ↓ *	RT-qPCR; WB	[49]
**Ageing and Senescence**					
Knee cartilage	Ageing	Ageing cartilage (*n* = 9) vs. normal cartilage (*n* = 9)	SESN2 ↓ * SESN3 ↓ *	IHC	[50]
Skeletal muscle (human)	Ageing	Older donors (*n* = 4) vs. younger donors (*n* = 3)	SESN1 ↓ (NR)	WB	[51]
Serum	Ageing	65–80 years old (*n* = 40) vs. 18–25 years old donors (*n* = 40)	SESN1 ↓ *	ELISA	[51]
Skeletal muscle (vastus lateralis)	Ageing	Old (*n* = 26) vs. young (*n* = 31)	SESN1 ↓ * SESN3 ↓ *	WB	[52]
HUVEC	Endothelial cell senescence	Senescent cells (*n* = 3) vs. younger control (*n* = 3)	SESN3 ↓ *	RT-qPCR	[53]
PBMC	Medically induced menopause (fertility treatment model)	Progesterone therapy (*n* = 13) vs. basal condition (*n* = 14)	SESN2 ↑ *	RT-qPCR	[54]
Peripheral blood CD8+ T cells	Age-associated T-cell senescence	CD27-CD28-CD8+ (senescent) (*n* = 10) vs. CD27+CD28+CD8+ (naïve) (*n* = 10)	SESN1 ↑ *	FC	[55]
Peripheral blood CD8+ T cells	Age-associated T-cell senescence	CD27-CD28-CD8+ (senescent) (*n* = 4) vs. CD27+CD28+CD8+ (naïve) (*n* = 4)	SESN2 ↑ *	WB	[55]
Peripheral blood CD8+ T cells	Age-associated T-cell senescence	CD27+CD28-CD8+ (intermediate) (*n* = 4) vs. CD27+CD28+CD8+ (naïve) (*n* = 4)	SESN2 ↑ *	WB	[55]
Peripheral blood CD8+ T cells	Age-associated T-cell senescence	Old (>65 years, *n* = 4) vs. young (<35 years, *n* = 5)	SESN2 ↑ *	FC	[55]
Peripheral blood CD8+ T cells	Age-associated T-cell senescence	Terminal effector cells (*n* = 6) vs. naïve T cells (*n* = 6)	SESN2 ↑ (NR)	scRNA-seq	[55]
Peripheral blood CD4+ T cells	Age-associated T-cell senescence	CD27-CD28-CD4+ (senescent) (*n* = 4) vs. CD27+CD28+CD4+ (non-senescent) (*n* = 4)	SESN1 ↑ * SESN2 ↑ * SESN3 ↑ *	FC	[56]
Peripheral blood CD4+ T cells	Ageing	70–85 years (*n* = 3) vs. 20–35 years (*n* = 3)	SESN1 ↑ * SESN2 ↑ * SESN3 ↑ *	WB	[56]
Peripheral blood CD4+ T cells	X-ray irradiation	8 h post-X-ray (*n* = 3) vs. control (*n* = 3)	SESN1 ↑ * SESN2 ↑ *	FC	[56]
**Neurodegeneration/Neurological disease**					
Brain (hippocampus slices)	Temporal lobe epilepsy	TLE patient samples (*n* = 7) vs. control (*n* = 8)	SESN3 ↑ *	IF	[57]
Brain (substantia nigra)	Parkinson’s disease	PD (*n* = 5) vs. control (*n* = 4)	SESN2 ↑ *	WB; IHC	[58]
Brain (substantia nigra and putamen)	Parkinson’s disease	PD (*n* = 3) vs. control (*n* = 3)	SESN3 – (NS)	WB	[59]
Skeletal muscle (multiple)	Amyotrophic lateral sclerosis	ALS (MA *n* = 3; RT-qPCR *n* =7) vs. control (MA *n* = 3; RT-qPCR *n* = 7)	SESN3 ↑ *	MA; RT-qPCR	[60]
**Respiratory Disease**					
Lung	COPD (GOLD stage IV)	Advanced COPD (*n* = 12) vs. healthy donor (*n* = 11)	SESN2 ↑ *	WB	[61]
Polyp tissue homogenate	Nasal polyposis	NP patients (*n* = 50) vs. control (*n* = 40)	SESN2 ↓ (NS)	ELISA	[62]
Sputum supernatant and sputum cell pellet	Asthma	Severe asthma (*n* = 35) vs. mild to moderate asthma (*n* = 25)	SESN2 ↑ *	ELISA	[63]
**Degenerative**					
Anterior lens capsule	Cataract	Cataract patients (RT-qPCR *n* = 25; WB = 3) vs. control (RT-qPCR *n* = 25; WB = 3)	SESN2 ↓ *	RT-qPCR; WB	[64]
Articular cartilage	Osteoarthritis	Damaged area (*n* = 10) vs. relatively intact area (*n* = 10)	SESN2 ↓ *	IHC; WB	[65]
Gastric mucosal tissue	Gastric intestinal metaplasia	GIM (*n* = 15) vs. control (*n* = 15)	SESN2 ↑ *	RT-qPCR; IHC	[66]
Knee cartilage	Osteoarthritis	Osteoarthritis (*n* = 25) vs. normal cartilage (*n* = 25)	SESN2 ↓ *	RT-qPCR	[67]
Knee cartilage	Osteoarthritis	Osteoarthritis (*n* = 6) vs. normal cartilage (*n* = 6)	SESN1 ↓ * SESN2 ↓ * SESN3 ↓ *	RT-qPCR	[50]
Knee cartilage	Osteoarthritis	Osteoarthritis (*n* = 7) vs. normal cartilage (*n* = 9)	SESN1 ↓ * SESN2 ↓ * SESN3 ↓ *	IHC	[50]
Ligamentum flavum	Ligamentum flavum hypertrophy	LFH cells (cells = 25,187) vs. non-LFH cells (cells = 13,747)	SESN2 ↓ (NR)	scRNA-seq	[68]
Ligamentum flavum	Ligamentum flavum hypertrophy	LFH (*n* = 6) vs. non-LFH (*n* = 6)	SESN2 ↓ *	IHC	[68]
Ligamentum flavum	Ligamentum flavum hypertrophy	LFH (*n* = 6) vs. non-LFH (*n* = 6)	SESN2 ↓ *	WB	[68]
Liver	Liver cirrhosis	Cirrhotic liver (*n* = 6) vs. normal liver (*n* = 6)	SESN2 ↓ *	IHC	[69]
Liver	Liver fibrosis	Fibrotic liver (*n* = 5) vs. normal liver (*n* = 5)	SESN2 ↓ *	WB	[69]
Nucleus pulposus (lumbar disc)	Intervertebral disc degeneration	IDD NuP tissues (*n* = 18) vs. normal NuP tissues (*n* = 18)	SESN1 ↓ * SESN2 ↓ * SESN3 ↓ *	RT-qPCR; WB; IHC (SESN2)	[70]
Primary fibroblasts (kEDS)	Kyphoscoliotic Ehlers–Danlos syndrome	kEDS fibroblasts (*n* = 3) vs. control (*n* = 3)	SESN2 ↑ *	RT-qPCR	[71]
**Physiological/Experimental condition**					
Skin (ex vivo explants)	UVB exposure	UVB exposed (*n* = NR) vs. non-irradiated control (*n* = NR)	SESN2 ↑ (NR)	WB	[72]
Skeletal muscle (vastus lateralis)	Acute resistance exercise	2 h post-acute RE (*n* = 9) vs. pre-exercise (*n* = 9)	SESN2 ↑ *	RT-qPCR	[73]
Skeletal muscle (vastus lateralis)	Resistance training	12-week RET (*n* = 10) vs. pre-training (*n* = 10)	SESN1 ↑ *	WB	[73]
Skeletal muscle (vastus lateralis)	Muscle disuse in middle-aged men (45–60 years)	14-day immobilisation (*n* = 25) vs. pre-immobilization (*n* = 25)	SESN2 ↓ *	WB	[74]
Skeletal muscle (vastus lateralis)	Ageing (dietary protein intervention)	2x RDA (*n* = 12) vs. RDA (*n* = 14)	SESN1 – SESN2 – SESN3 – (NS)	RT-qPCR	[75]
Skeletal muscle (vastus lateralis)	Acute MPC ingestion in middle-aged men (45–60 years)	3.5 h post-20 g MPC (*n* = 8) vs. baseline (*n* = 8)	SESN2 – (NS)	WB	[76]
BJ fibroblasts	Oxidative stress (nanomaterial exposure model)	5 h post-ZnO nanomaterials (50 μM) (*n* = 3) vs. control (*n* = 3)	SESN1 ↑ * SESN2 ↑ *	RT-qPCR	[77]
Endothelial progenitor cells	Hormone stimulation (Angiotensin II)	Ang II-treated EPCs (*n* = 3) vs. control (*n* = 3)	SESN2 ↑ (NR)	RT-qPCR; WB	[78]
PBMC	Endometrial cancer patients undergoing radiotherapy	1–2 fractions of RT (*n* = 10) vs. pre-RT control (*n* = 10)	SESN1 ↑ *	RT-qPCR	[79]
PBMC	Endometrial cancer patients undergoing radiotherapy	25 fractions of RT (*n* = 10) vs. pre-RT control (*n* = 10)	SESN1 ↓ *	RT-qPCR	[79]
Peripheral blood monocytes	Surgery and anaesthesia	Post-operation (*n* = 3) vs. pre-operation (*n* = 3)	SESN1 ↑ *	RT-qPCR	[80]
Peripheral blood leukocytes	Ionising radiation exposure (healthy donors)	Irradiated blood (*n* = 3) vs. control (*n* = 3)	SESN1 ↑ (NR)	MA	[81]
Peripheral blood leukocytes	Cancer patients undergoing radiotherapy	Mid-RT (*n* = 6) vs. pre-treatment baseline (*n* = 6)	SESN1 ↑ *	RT-qPCR	[82]
Peripheral blood leukocytes	Cancer patients undergoing radiotherapy	18 h post-RT (*n* = 6) vs. pre-treatment baseline (*n* = 6)	SESN1 ↑ *	RT-qPCR	[82]
Mesenchymal stem cells	Ultra-endurance cycling athletes training	Post-training (*n* = 8) vs. pre-training period (*n* = 8)	SESN1 ↑ *	RT-qPCR	[83]
Skeletal muscle cells (cultured with participant sera)	Ultra-endurance cycling athletes training	Post-training serum (*n* = 8) vs. pre-training serum (*n* = 8)	SESN1 ↑ * SESN2 ↑ *	WB	[83]
PBMC-derived RNA	Cancer patients undergoing radiotherapy	Immediately and 1-month post-RT (*n* = 23) vs. pre-treatment baseline (*n* = 23)	SESN1 ↓ *	RT-qPCR	[84]

**Table 2:** Mouse studies reporting Sestrin expression changes. Summary of published studies reporting Sesn1, Sesn2, or Sesn3 expression changes measured in mouse tissue biopsies or primary mouse cells across multiple disease models and experimental conditions. “Test (*n*) vs. Comparator (*n*)” indicates the groups used for Sestrin comparison. “Sestrin change” denotes the direction of expression relative to the study-defined control group. Symbols indicate the reported direction of change (↑, upregulation; ↓, downregulation; –, no change). Statistical significance is indicated by * where *p* < 0.05 (NS, not significant; NR, not reported). “Assay” denotes the method used to measure Sestrin expression. Abbreviations: AB, Aortic banding; AD, Alzheimer’s disease; AKI, Acute kidney injury; APAP, Acetaminophen (paracetamol); ApoE-/-, Apolipoprotein E knockout mouse; AS, Atherosclerosis; BDL, Bile duct ligation; BLM, Bleomycin; BSNP, β-glucan-stimulated nanoparticle (trained immunity stimulus); CLP, Cecal ligation and puncture; CRS, Chronic restraint stress; CSE, Cigarette smoke extract; dB SPL, Decibel sound pressure level; db/db, Leptin receptor-deficient diabetic mouse; db/m, Heterozygous non-diabetic control mouse; DKD, Diabetic kidney disease; DOX, Doxorubicin; DSS, Dextran sulphate sodium; EAE, Experimental autoimmune encephalomyelitis; ELISA, Enzyme-linked immunosorbent assay; ER, Endoplasmic reticulum; FC, Flow cytometry; HFD, High-fat diet; HFpEF, Heart failure with preserved ejection fraction; IF, Immunofluorescence; IHC, Immunohistochemistry; KK-Ay, Obese diabetic KK-Ay mouse model; LAD, Left anterior descending coronary artery; LFD, Low-fat diet; LPS, Lipopolysaccharide; MASLD, Metabolic dysfunction-associated steatotic liver disease; MCAO/R, Middle cerebral artery occlusion/reperfusion; MCD, Methionine-choline deficient diet; MI, Myocardial infarction; NAFLD, Non-alcoholic fatty liver disease; NASH, Non-alcoholic steatohepatitis; NIAAA, National Institute on Alcohol Abuse and Alcoholism; NK cells, Natural killer cells; PBS, Phosphate-buffered saline; PiZ, Alpha-1 antitrypsin Z mutation transgenic mouse; RT-qPCR, Reverse transcription quantitative polymerase chain reaction; SNI, Spared nerve injury; STZ, Streptozotocin; T2DM, Type 2 diabetes mellitus; UVB, Ultraviolet B radiation; WB, Western blot; WT, Wild type.

Tissue	Condition	Test (*n*) vs. Comparator (*n*)	Sestrin Change	Assay	Ref.
**Diabetes**					
Heart	Type 1 diabetes (streptozotocin-induced)	6-month post-STZ-diabetes (*n* = 5) vs. 6-month control (*n* = 5)	Sesn2 ↓ *	RT-qPCR; WB	[85]
Heart	Type 1 diabetes (streptozotocin-induced)	2-month post-STZ-diabetes (*n* = 3) vs. control (*n* = 3)	Sesn2 ↑ *	WB	[86]
Kidney	Diabetic kidney disease (KK-Ay model)	DKD (*n* = 4) vs. control (*n* = 4)	Sesn2 ↓ *	WB	[87]
Kidney	Diabetic nephropathy (HFD + STZ model)	T2DM (*n* = 10) vs. control (*n* = 10)	Sesn2 ↓ *	RT-qPCR; WB	[88]
Kidney (renal cortex)	Type 2 diabetes (db/db model)	db/db (*n* = 3) vs. db/m (*n* = 3)	Sesn2 ↓ *	WB	[89]
Liver	Type 2 diabetes (db/db model)	db/db (*n* = 4–5) vs. WT control (*n* = 4–5)	Sesn2 ↓ *	RT-qPCR	[90]
Liver	Type 1 diabetes (streptozotocin-induced)	2-week post-STZ-diabetes (*n* = 4–5) vs. control (*n* = 4–5)	Sesn2 ↓ (NS)	RT-qPCR	[90]
**Metabolic**					
Brain (micropunch dissection of hypothalamus)	HFD-induced obesity	14-week HFD (*n* = 6) vs. control (*n* = 6)	Sesn2 ↓ *	IF; WB	[91]
Brain (micropunch dissection of hypothalamus)	Genetic obesity (db/db model)	db/db mice (*n* = 6) vs. db/m mice (*n* = 6)	Sesn2 ↓ *	IF; WB	[91]
Brown adipose tissue	HFD-induced obesity	3-week HFD (*n* = 7) vs. control (*n* = 7)	Sesn2 ↑ *	WB	[92]
Heart (left ventricle)	HFD-induced obesity	16-week HFD (*n* = 6) vs. control (*n* = 6)	Sesn2 ↓ *	WB	[34]
Liver	HFD-induced obesity	8-week HFD (*n* = 5) vs. control (*n* = 5)	Sesn2 ↑ *	WB	[93]
Liver	HFD-induced MASLD	16-week HFD (*n* = 4) vs. control (*n* = 4)	Sesn2 ↓ *	WB	[94]
Liver	MCD-induced NASH	MCD group (*n* = 4–6) vs. control (*n* = 4–6)	Sesn2 ↑ (NS)	WB	[95]
Liver	Obesity-related NAFLD	12-week HFD (*n* = 9) vs. control (*n* = 9)	Sesn2 ↓ *	RT-qPCR	[96]
Liver	Obesity-related NAFLD	12-week HFD (*n* = 6) vs. control (*n* = 6)	Sesn2 – (NS)	WB	[96]
Liver	Western diet-induced NAFLD	8-week Western diet (*n* = 4) vs. control diet (*n* = 4)	Sesn1 ↓ * Sesn2 ↓ * Sesn3 ↓ *	WB	[97]
Liver	Alcohol-induced hepatic steatosis (Lieber-DeCarli diet)	Ethanol-fed mice (*n* = 3) vs. control mice (*n* = 3)	Sesn1 ↓ * Sesn2 ↓ * Sesn3 ↓ *	RT-qPCR; WB	[98]
Placenta	Maternal obesity (HFD model)	≥6-week HFD dams (*n* = 14) vs. LFD dams (*n* = 14)	Sesn2 ↑ *	RT-qPCR	[99]
Skeletal muscle (quadriceps)	HFD-induced insulin resistance	12-week HFD (*n* = 6) vs. control (*n* = 6)	Sesn3 ↓ *	RT-qPCR; WB	[100]
Skeletal muscle (quadriceps)	HFD-induced lipid disorder	14-week HFD (*n* = 6) vs. control (*n* = 6)	Sesn2 ↑ *	WB	[101]
Skeletal muscle (vastus lateralis)	HFD-induced obesity	12-week HFD (*n* = 6) vs. control (*n* = 6)	Sesn2 ↑ *	WB	[102]
Subcutaneous white adipose tissue	HFD-induced obesity	3-week HFD (*n* = 3) vs. sedentary control (*n* = 3)	Sesn2 – (NS)	WB	[92]
Embryonic fibroblasts	Metabolic stress (glucose limitation)	1 mM glucose (*n* = 3) vs. 25 mM glucose (*n* = 3)	Sesn2 ↑ *	RT-qPCR	[103]
Hepatocytes (PiZ)	ER storage disorder (α1-antitrypsin deficiency)	PiZ hepatocytes (*n* = 3) vs. WT hepatocytes (*n* = 3)	Sesn2 ↑ *	RT-qPCR	[71]
**Cardiovascular**					
Aorta	Atherosclerosis (ApoE-/- mouse model)	AS model (*n* = 5) vs. control (*n* = 5)	Sesn1 ↑ *	WB	[104]
Brain (prefrontal lobe)	Myocardial infarction (LAD ligation model)	MI (IF *n* = 3; WB *n* = 5) vs. sham control (IF *n* = 3; WB *n* = 5)	Sesn2 ↑ *	IF; WB	[105]
Heart	Heart failure with preserved ejection fraction (HFpEF)	HFpEF model (*n* = 6) vs. control (*n* = 6)	Sesn3 ↑ *	WB	[106]
Heart	Pressure overload (aortic banding)	2 and 4 weeks post-AB surgery vs. sham control (*n* = 6/group)	Sesn2 ↑ *	RT-qPCR; WB	[107]
Heart	Diabetic cardiomyopathy (KK-Ay mice, HFD-induced)	KK-Ay mice (*n* = 15) vs. control (*n* = 15)	Sesn2 ↑ *	WB	[108]
Heart	Doxorubicin cardiotoxicity	24 h after DOX treatment (*n* = 3) vs. PBS-treated mice (*n* = 3)	Sesn1 ↑ * Sesn2 ↑ *	WB	[109]
Heart	Diabetic cardiomyopathy (KK-Ay mice, HFD-induced)	KK-Ay mice (*n* = NR) vs. control (*n* = NR)	Sesn2 ↑ *	WB	[110]
Lung	Heart failure with preserved ejection fraction (HFpEF)	HFpEF model (*n* = 6) vs. control (*n* = 6)	Sesn3 ↑ *	WB	[106]
Macrophages (from infarcted myocardium)	Myocardial infarction (coronary artery ligation)	3 and 5 days post MI (*n* = 3) vs. sham control (*n* = 3)	Sesn2 ↑ *	WB	[111]
**Inflammatory/Infectious**					
Brain (cerebral cortex)	Sepsis-induced brain injury (CLP model)	24 h post-CLP (*n* = 4) vs. sham control (*n* = 4)	Sesn2 ↑ *	WB	[112]
Brain (hippocampus)	Sepsis-associated encephalopathy (CLP model)	2–16 h post-CLP (*n* = 4) vs. sham control (*n* = 4)	Sesn2 ↑ *	WB	[113]
Brain (hippocampus)	Chronic inflammation model (multiple intraperitoneal LPS injections)	5-day LPS treatment (*n* = 5) vs. control (*n* = 5)	Sesn2 ↑ *	WB	[114]
Colon	Experimental autoimmune encephalomyelitis (multiple sclerosis model)	EAE (*n* = 5–7) vs. control (*n* = 5–7)	Sesn3 ↓ *	ELISA	[115]
Heart	LPS-induced inflammatory cardiomyopathy model	24 h LPS treatment (*n* = 4) vs. control (*n* = 4)	Sesn2 ↑ *	WB	[116]
Liver	Sepsis-induced liver injury (CLP model)	CLP (*n* = 6) vs. sham control (*n* = 6)	Sesn2 ↓ *	IHC; WB	[117]
Alveolar macrophages	LPS-induced acute lung injury	16 h LPS treatment (*n* = 3) vs. control (*n* = 3)	Sesn2 ↑ *	WB	[118]
Colon LP macrophages	DSS-induced acute colitis	Acute colitis (*n* = 6) vs. control (*n* = 6)	Sesn2 ↑ * Sesn3 ↑ *	RT-qPCR	[119]
Colon LP neutrophils	DSS-induced acute colitis	Acute colitis (*n* = 6) vs. control (*n* = 6)	Sesn1 ↑ *	RT-qPCR	[119]
Colon LP CD4+ T cells	DSS-induced acute colitis	Acute colitis (*n* = 6) vs. control (*n* = 6)	Sesn1 ↑ * Sesn2 ↑ *	RT-qPCR	[119]
Colon LP NK cells	DSS-induced acute colitis	Acute colitis (*n* = 6) vs. control (*n* = 6)	Sesn1 ↓ *	RT-qPCR	[119]
Peritoneal macrophages	Trained immunity (BSNP-treated mice)	BSNP-treated mice macrophages (*n* = NR) vs. control macrophages (*n* = NR)	Sesn1 ↑ (NR)	PCR Array	[120]
Splenic dendritic cells	Sepsis (CLP model)	12–24 h post-CLP (*n* = 3) vs. sham control (*n* = 3)	Sesn2 ↑ *	WB	[121]
Splenic dendritic cells	LPS-induced inflammation	3–24 h LPS treatment (*n* = 3) vs. control (*n* = 3)	Sesn2 ↑ *	WB	[121]
Splenic dendritic cells	LPS-induced inflammation	6–48 h LPS treatment (*n* = 1) vs. control (*n* = 1)	Sesn2 ↑ (NR)	WB	[122]
**Ageing and Senescence**					
Heart	Ageing	12- and 24-month vs. 4-month (*n* = 4/group)	Sesn2 ↓ *	WB	[123]
Heart (left ventricle)	Ageing	22-month vs. 4-month (*n* = 4–5/group)	Sesn2 ↓ (NR)	WB	[124]
Nucleus pulposus (lumbar disc)	Ageing (6–36 months)	12-, 24-, and 36-month vs. 6-month (*n* = 5/group)	Sesn3 ↓ *	IHC	[125]
Prostate	Ageing	13- and 24-month (*n* = 4) vs. 3-month (*n* = 4)	Sesn2 ↓ *	WB	[126]
Skeletal muscle	Ageing	24-month (*n* = 8) vs. 4-month (*n* = 8)	Sesn1 ↓ *	WB	[127]
Skeletal muscle (gastrocnemius)	Age-related sarcopenia	20-month (*n* = 3) vs. 6-month (*n* = 3)	Sesn1 ↓ * Sesn2 ↓ *	WB	[128]
Skeletal muscle (gastrocnemius)	Age-related sarcopenia	18-month (*n* = 8) vs. 2-month (*n* = 8)	Sesn2 ↑ *	WB	[129]
Skeletal muscle (gastrocnemius)	Ageing	24-month (*n* = 3) vs. 3-month (*n* = 3)	Sesn2 ↓ *	WB	[130]
Skeletal muscle (gastrocnemius)	Ageing	24–25-month (*n* = 6) vs. 2–3-month (*n* = 6)	Sesn2 ↓ *	IHC	[131]
Skeletal muscle (quadriceps)	Age-related sarcopenia	22-month sedentary mice (*n* = 3) vs. 8-week mice (*n* = 3)	Sesn1 ↓ (NR)	WB	[132]
CD4+ T cells (spleen)	Ageing	20-month (*n* = 3) vs. 2-month (*n* = 3)	Sesn1 ↑ * Sesn2 ↑ * Sesn3 ↑ *	FC	[56]
**Ischemic/Injury Models**					
Brain (cerebral cortex)	Cerebral ischemia–reperfusion injury	30 min ischemia + 24 h reperfusion (*n* = 10) vs. sham control (*n* = 10)	Sesn2 ↓ *	WB	[133]
Brain (hippocampus)	Global cerebral ischemia with STZ-induced diabetes	15 min global ischemia + STZ-diabetes (*n* = 27) vs. control (*n* = 10)	Sesn3 ↑ *	WB	[134]
Brain (ischemic cortical penumbra)	Cerebral ischemia–reperfusion injury (MCAO/R model)	1 h ischemia + 24–48 h reperfusion (*n* = 6) vs. sham control (*n* = 6)	Sesn2 ↑ *	WB	[135]
Brain (ischemic cortical penumbra)	Cerebral ischemia–reperfusion injury (MCAO/R model)	1 h ischemia + 72 h reperfusion (*n* = 6) vs. sham control (*n* = 6)	Sesn2 ↑ *	RT-qPCR	[136]
Brain (ischemic cortical penumbra)	Cerebral ischemia–reperfusion injury (MCAO/R model)	1 h ischemia + 72 h reperfusion (*n* = 5) vs. sham control (*n* = 5)	Sesn2 ↑ *	WB	[137]
Cochlea	Noise-induced hearing loss	Noise exposure (120 dB SPL, 4 h) (*n* = 3) vs. control (*n* = 3)	Sesn2 ↑ *	WB	[138]
Colon	Alcohol-induced intestinal injury (NIAAA model)	Alcohol (*n* = 3) vs. control (*n* = 3)	Sesn2 ↓ *	WB	[139]
Corneal epithelium	Corneal epithelial wound healing	Wounded cornea (*n* = NR) vs. non-wounded control (*n* = NR)	Sesn2 ↓ (NR)	WB	[140]
Heart (left ventricle)	Myocardial ischemia/reperfusion injury	45 min ischemia + 24 h reperfusion (*n* = 6) vs. sham control (*n* = 6)	Sesn2 ↑ *	WB	[141]
Kidney	Iodinated contrast media-induced acute kidney injury	24 h post-AKI (*n* = 7) vs. control (*n* = 7)	Sesn2 ↓ *	WB	[142]
Liver	Hepatic ischemia–reperfusion injury	90 min ischemia + 6 h reperfusion (*n* = 6) vs. sham control (*n* = 6)	Sesn2 ↑ (NR)	RT-qPCR	[143]
Liver	Acetaminophen-induced liver injury	APAP (300 mg/kg, 6 h) (*n* = 6) vs. control (*n* = 6)	Sesn2 ↑ *	WB	[144]
Liver	Acetaminophen-induced liver injury	APAP (300 mg/kg, 24 h) (*n* = NR) vs. control (*n* = NR)	Sesn2 ↓ *	WB	[145]
Liver	Cholestatic liver injury (bile duct ligation)	BDL (*n* = 5–10) vs. sham control (*n* = 5–10)	Sesn1 ↑ (NR)	WB	[146]
Liver	Cholestatic liver injury (bile duct ligation)	BDL (*n* = 6) vs. sham control (*n* = 5)	Sesn2 ↑ *	RT-qPCR; WB	[147]
Lung	Pulmonary fibrosis (bleomycin model)	1-week post-BLM (*n* = 6) vs. control (*n* = 6)	Sesn3 ↑ *	WB	[148]
Lung	Pulmonary fibrosis (bleomycin model)	2-week post-BLM (*n* = 6) vs. control (*n* = 6)	Sesn3 ↑ *	WB	[148]
Lung	Pulmonary fibrosis (bleomycin model)	4-week post-BLM (*n* = 3) vs. control (*n* = 3)	Sesn2 ↓ *	WB	[149]
Lung	Pulmonary fibrosis (bleomycin model)	4-week post-BLM (*n* = 10) vs. control (*n* = 10)	Sesn2 ↓ *	IHC	[150]
Lumbar dorsal root ganglia	Neuropathic pain (Spared Nerve Injury model)	SNI-operated mice (*n* = 4–8) vs. control (*n* = 4–8)	Sesn2 ↑ *	RT-qPCR	[151]
Pancreas	Caerulein-induced acute pancreatitis	24 h after caerulein treatment (*n* = 7) vs. NaCl control (*n* = 7)	Sesn2 ↑ (NR)	RT-qPCR	[152]
Sciatic nerve	Neuropathic pain (Spared Nerve Injury model)	1–3 weeks post-SNI (*n* = 6–10) vs. control (*n* = 6–10)	Sesn2 ↑ *	WB	[151]
Skin	Deep second-degree burn	12 h–2 days after burn wound (*n* = 5) vs. control (*n* = 5)	Sesn2 ↑ *	WB	[153]
**Neurodegeneration/Neurological disease**					
Brain (cerebral cortex/prefrontal cortex)	Alzheimer’s disease (APPswe/PSEN1dE9 model)	9-month transgenic AD mice (*n* = 7) vs. control (*n* = 5)	Sesn2 ↓ *	WB	[154]
Brain (cerebral cortex)	Alzheimer’s disease (APPswe/PSEN1dE9 model)	12-month transgenic AD mice (*n* = 3) vs. control (*n* = 3)	Sesn2 ↑ *	WB	[155]
Brain (cerebral cortex)	Alzheimer’s disease (APPswe/PSEN1dE9 model)	Transgenic AD mice (*n* = 3) vs. control (*n* = 3)	Sesn1 ↑ *	RT-qPCR; WB	[156]
Brain (cerebral cortex)	Accelerated ageing/axonal degeneration model	Foxo1/3/4 KO mice (*n* = 3) vs. WT control (*n* = 3)	Sesn3 ↓ *	WB	[157]
**Degenerative**					
Skeletal muscle (diaphragm, gastrocnemius, and tibialis anterior)	Mdx mice (Duchenne muscular dystrophy model)	Mdx mice (*n* = 5) vs. control mice (*n* = 5)	Sesn2 ↑ *	WB	[158]
Osteoblasts	Osteogenesis imperfecta (Amish OI model)	Amish osteoblasts (*n* = 3) vs. control (*n* = 3)	Sesn2 ↑ *	RT-qPCR	[71]
**Musculoskeletal Remodelling/Atrophy**					
Skeletal muscle (gastrocnemius)	Muscle disuse via immobilisation	2-week immobilisation (*n* = 3–4) vs. sedentary control (*n* = 3–4)	Sesn2 ↓ *	WB	[159]
Skeletal muscle (gastrocnemius)	Denervation-induced muscle atrophy	2-week post-denervation (*n* = 6) vs. sham control (*n* = 6)	Sesn2 ↑ *	WB	[160]
Skeletal muscle (gastrocnemius)	Denervation-induced muscle atrophy	4-week post-denervation (*n* = 6) vs. sham control (*n* = 6)	Sesn2 – (NS)	WB	[160]
Skeletal muscle (gastrocnemius)	Denervation-induced muscle atrophy	2-week post-denervation (*n* = 6) vs. sham control (*n* = 6)	Sesn2 ↑ *	WB	[161]
Skeletal muscle (gastrocnemius)	Denervation-induced muscle atrophy	4-week post-denervation (*n* = 6) vs. sham control (*n* = 6)	Sesn2 – (NS)	WB	[161]
Skeletal muscle (gastrocnemius)	Muscle disuse via immobilisation	1-week immobilisation (*n* = 5) vs. control (*n* = 5)	Sesn1 ↓ *	WB	[162]
Skeletal muscle (gastrocnemius)	Muscle disuse via immobilisation	1-week immobilisation (*n* = 5) vs. control (*n* = 5)	Sesn2 ↑ *	WB	[162]
Skeletal muscle (longissimus dorsi)	Microgravity-induced muscle adaptation (spaceflight model)	Spaceflight mice (*n* = 5) vs. ground control (*n* = 5)	Sesn1 ↑ *	RT-qPCR	[163]
Skeletal muscle (soleus)	Muscle disuse via immobilisation	24 h immobilisation (*n* = 7) vs. contralateral non-immobilised (*n* = 7)	Sesn1 ↓ *	WB	[164]
Skeletal muscle (tibialis anterior)	Muscle disuse via immobilisation	10-day immobilisation (*n* = 5) vs. basal control (*n* = 5)	Sesn1 ↓ *	WB	[127]
**Physiological/Experimental conditions**					
Brain (hippocampus)	Cigarette smoke extract exposure	CSE-exposed group (*n* = 3) vs. control (*n* = 3)	Sesn2 ↑ *	WB	[165]
Brown adipose tissue	Arsenite exposure	10 mg/kg arsenite (*n* = 3) vs. control (*n* = 3)	Sesn2 ↓ *	RT-qPCR; WB	[166]
Brown adipose tissue	HFD and exercise intervention	HFD + exercise; control + exercise vs. sedentary control (*n* = 7/group)	Sesn2 ↑ *	WB	[92]
Liver	ER stress (tunicamycin model)	Tunicamycin-injected (*n* = 1) vs. control (*n* = 1)	Sesn2 ↑ (NR)	WB	[167]
Lung	Acute restraint stress	4 h restraint stress (*n* = 5) vs. control (*n* = 4)	Sesn1 ↑ *	RT-qPCR	[168]
Skeletal muscle (extensor digitorum longus, gastrocnemius, and tibialis anterior)	Ageing and Training	24-month exercise (*n* = 3) vs. 24-month rest (*n* = 3)	Sesn2 ↑ *	WB	[130]
Skeletal muscle (gastrocnemius)	Chronic restraint stress (CRS)	CRS (*n* = 6) vs. control (*n* = 6)	Sesn1 ↓ * Sesn2 ↓ * Sesn3 ↓ *	RT-qPCR	[169]
Skeletal muscle (gastrocnemius)	Chronic restraint stress (CRS)	CRS (*n* = 3) vs. control (*n* = 3)	Sesn2 ↓ *	WB	[169]
Skeletal muscle (plantaris/gastrocnemius)	Acute ethanol exposure	Alcohol injection (*n* = 8) vs. saline (*n* = 8)	Sesn2 ↓ *	WB	[170]
Skeletal muscle (quadriceps)	Exercise training (with HFD)	HFD exercise (*n* = 3) vs. HFD control (*n* = 3)	Sesn2 ↑ *	WB	[171]
Skeletal muscle (quadriceps)	Exercise training (with HFD)	HFD exercise (*n* = 4) vs. HFD control (*n* = 4)	Sesn2 ↑ * Sesn3 ↑ *	WB	[172]
Skeletal muscle (quadriceps)	Exercise training	Exercise (*n* = 4) vs. control (*n* = 4)	Sesn2 ↑ * Sesn3 ↑ *	WB	[172]
Skeletal muscle (quadriceps)	Fasting	24 h fasting (*n* = 8) vs. control (*n* = 8)	Sesn1 ↑ *	RT-qPCR	[173]
Skin	Chronic UVB exposure (3 times per week, 23 weeks)	UVB treatment (*n* = NR) vs. control (*n* = NR)	Sesn2 ↑ (NR)	WB	[174]
Subcutaneous white adipose tissue	HFD and exercise intervention	HFD + exercise; normal diet + exercise (*n* = 3/group) vs. sedentary control (*n* = 3)	Sesn2 ↑ *	WB	[92]
Aortic macrophages and monocytes	Atherosclerosis (ApoE-/- mouse model)	Aerobic exercise group (*n* = 7) vs. control group (*n* = 7)	Sesn1 ↑ *	RT-qPCR	[175]

**Table 3:** Rat studies reporting Sestrin expression changes. Summary of published studies reporting Sesn1, Sesn2, or Sesn3 expression changes measured in rat tissue biopsies or primary rat cells across multiple disease models and experimental conditions. “Test (*n*) vs. Comparator (*n*)” indicates the groups used for Sestrin comparison. “Sestrin change” denotes the direction of expression relative to the study-defined control group. Symbols indicate the reported direction of change (↑, upregulation; ↓, downregulation; –, no change). Statistical significance is indicated by * where *p* < 0.05 (NS, not significant; NR, not reported). “Assay” defines the method used to measure Sestrin expression. Abbreviations: 4-AP, 4-Aminopyridine; Aβ25–35, Amyloid-β peptide fragment 25–35; ADR, Adriamycin; AMI, Acute myocardial infarction; AngII, Angiotensin II; BiC, Bicuculline; BLM, Bleomycin; CUS, Chronic unpredictable stress; EPC, Endothelial progenitor cell; HFD, High-fat diet; IF, Immunofluorescence; IHC, Immunohistochemistry; LPS, Lipopolysaccharide; MAL, Malonic acid; MCAO/R, Middle cerebral artery occlusion with reperfusion; MIA, Monosodium iodoacetate; NaAsO_2_, Sodium arsenite; PQ, Paraquat; RT-qPCR, Reverse transcription quantitative polymerase chain reaction; STZ, Streptozotocin; T3, Triiodothyronine; WB, Western blot; ZDF, Zucker diabetic fatty.

Tissue	Condition	Test (*n*) vs. Comparator (*n*)	Sestrin change	Assay	Ref.
**Diabetes**					
Heart	Type 2 diabetes (ZDF model)	Zucker diabetic fatty rats (*n* = 6) vs. control (*n* = 6)	Sesn2 ↑ *	RT-qPCR	[176]
Heart	Type 1 diabetes with myocardial ischemia/reperfusion (STZ model)	30 min ischemia + 2 h reperfusion + STZ-diabetes (*n* = 8) vs. control (*n* = 8)	Sesn2 ↓ *	RT-qPCR	[177]
Kidney	Type 1 diabetes (STZ model)	STZ-diabetes (*n* = 6) vs. control (*n* = 6)	Sesn2 ↓ *	WB	[178]
Kidney	Diabetic nephropathy	STZ-diabetes (*n* = NR) vs. control (*n* = NR)	Sesn2 ↓ (NR)	WB	[179]
Kidney	Type 1 diabetes (STZ model)	STZ-diabetes (*n* = 2) vs. control (*n* = 2)	Sesn2 ↓ (NR)	WB	[180]
Retina	Diabetic retinopathy (STZ model)	Diabetes (*n* = 8) vs. control (*n* = 8)	Sesn2 ↓ *	WB	[181]
Retina	Diabetic retinopathy (STZ model)	Diabetic retinopathy (*n* = 5) vs. control (*n* = 5)	Sesn2 ↓ *	WB	[182]
**Metabolic**					
Cardiac fibroblasts	High glucose stimulation	High glucose (*n* = 3) vs. control (*n* = 3)	Sesn2 ↓ *	WB	[183]
Aorta	Diet-induced obesity	HFD (*n* = 6) vs. control (*n* = 6)	Sesn2 ↓ *	RT-qPCR; WB	[184]
Aorta	Diet-induced obesity with type 1 diabetes (STZ model)	HFD + STZ-diabetes (*n* = 6) vs. control (*n* = 6)	Sesn2 ↓ *	RT-qPCR; WB	[184]
Heart	Diet-induced obesity	HFD (*n* = 6) vs. control (*n* = 6)	Sesn2 ↓ * (qPCR—NS)	RT-qPCR; WB	[184]
Heart	Diet-induced obesity with type 1 diabetes (STZ model)	HFD + STZ-diabetes (*n* = 6) vs. control (*n* = 6)	Sesn2 ↓ *	RT-qPCR; WB	[184]
**Cardiovascular**					
Brain (hippocampus)	Cardiac arrest	8 min cardiac arrest (*n* = 6) vs. sham control (*n* = 6)	Sesn2 ↑ *	WB	[185]
Heart	Doxorubicin-induced cardiomyopathy	Doxorubicin (*n* = 6) vs. control (*n* = 4)	Sesn2 ↓ *	WB; IHC	[186]
Heart	Doxorubicin-induced cardiomyopathy	Doxorubicin (*n* = 3) vs. control (*n* = 3)	Sesn1 ↑ *	RT-qPCR	[186]
Heart	Acute myocardial infarction	1–14 days post-AMI (*n* = 3) vs. control (*n* = 3)	Sesn2 ↑ *	WB	[187]
Heart	Acute myocardial infarction	28 days post-AMI (*n* = 3) vs. control (*n* = 3)	Sesn2 – (NS)	WB	[187]
Endothelial progenitor cells	Hypertension model (AngII-induced EPC injury)	AngII-treated (*n* = NR) vs. control (*n* = NR)	Sesn2 ↓ (NR)	WB; IF	[188]
Neonatal cardiomyocytes	Cardiomyocyte hypertrophy (phenylephrine model)	24–48 h post-phenylephrine treatment (*n* = 5) vs. control (*n* = 5)	Sesn2 ↓ *	WB	[189]
**Inflammatory/Infectious**					
Heart	LPS-induced myocardial inflammation	6 h LPS (*n* = NR) vs. control (*n* = NR)	Sesn2 ↑ *	WB	[190]
Heart	LPS-induced myocardial inflammation	24 h LPS (*n* = 6) vs. control (*n* = 6)	Sesn2 ↓ *	RT-qPCR; WB	[191]
**Ischemic/Injury Models**					
Brain (cerebral cortex)	Cerebral ischemia–reperfusion injury (MCAO/R model)	1 h ischemia + 24 h reperfusion (*n* = 6) vs. sham control (*n* = 4)	Sesn2 ↑ *	RT-qPCR; WB	[192]
Brain (hippocampus)	Transient global cerebral ischemia	10 min global ischemia + 1–48 h reperfusion (*n* = 5–7) vs. sham control (*n* = 5–7)	Sesn2 ↑ *	WB	[193]
Brain (ischemic cortical penumbra)	Photothrombotic ischemic stroke	5 days after photothrombotic ischemia (*n* = 9) vs. sham control (*n* = 9)	Sesn2 ↑ (NR)	WB	[194]
Brain (ischemic cortical penumbra)	Photothrombotic ischemic stroke	1, 3, and 5 days after photothrombotic ischemia (*n* = 6) vs. sham control (*n* = 6)	Sesn2 ↑ *	WB	[195]
Brain (ischemic cortical penumbra)	Cerebral ischemia–reperfusion injury with hyperglycemia (MCAO/R model)	Hyperglycemia sham (*n* = 3) vs. normoglycemia sham (*n* = 3)	Sesn2 ↑ *	WB	[196]
Brain (ischemic cortical penumbra)	Cerebral ischemia–reperfusion injury with hyperglycemia (MCAO/R model)	1-day post-reperfusion hyperglycemia (*n* = 3) vs. 1-day post-reperfusion normoglycemia (*n* = 3)	Sesn2 ↑ *	WB	[196]
Heart	Myocardial ischemia/reperfusion injury	2 h ischemia 12 h reperfusion (RT-qPCR *n* = 5; WB *n* = 6) vs. control (RT-qPCR *n* = 5; WB *n* = 6)	Sesn1 ↓ *	RT-qPCR; WB	[197]
Kidney	Renal ischemia/reperfusion injury	30 min ischemia 48 h reperfusion (*n* = NR) vs. control (*n* = NR)	Sesn2 ↓ *	WB	[198]
Kidney	Renal ischemia/reperfusion injury	30 min ischemia 48 h reperfusion (*n* = 3) vs. control (*n* = 3)	Sesn2 ↓ *	WB	[199]
Kidney	Renal ischemia/reperfusion injury	60 min ischemia 3–48 h reperfusion (*n* = 5) vs. control (*n* = 5)	Sesn2 ↑ *	RT-qPCR	[200]
Kidney	Renal ischemia/reperfusion injury	60 min ischemia 3–72 h reperfusion (*n* = 6) vs. control (*n* = 6)	Sesn2 ↑ *	WB	[200]
Kidney	Adriamycin-induced nephropathy	8 days post-ADR (*n* = 6) vs. control (*n* = 6)	Sesn2 – (NS)	IHC	[201]
Kidney	Adriamycin-induced nephropathy	14 and 42 days post-ADR vs. control (*n* = 6/group)	Sesn2 ↓ *	IHC	[201]
Kidney	Paraquat-induced acute kidney injury	24 h post-PQ (*n* = 6) vs. control (*n* = 6)	Sesn2 ↑ *	RT-qPCR; WB	[202]
Liver	Sodium arsenite-induced liver injury	NaAsO_2_ 50 and 100 mg/L vs. control (*n* = 6/group)	Sesn2 ↑ *	RT-qPCR; IHC	[203]
Lung	Pulmonary fibrosis (bleomycin model)	4-week post-BLM (*n* = 6) vs. control (*n* = 6)	Sesn2 ↓ *	RT-qPCR	[204]
**Neurodegeneration/Neurological disease**					
Brain (hippocampus)	Sevoflurane-induced cognitive dysfunction	Sevoflurane (*n* = 6) vs. control (*n* = 6)	Sesn1 ↑ *	WB	[205]
Brain (striatum)	Malonic acid-induced Huntington’s disease model	MAL-treated rats (*n* = 3) vs. sham control (*n* = 3)	Sesn2 ↑ *	RT-qPCR	[206]
Cortical neurons	Alzheimer’s disease model (amyloid-β exposure)	Aβ25-35 (10 μM) (*n* = 3) vs. control (*n* = 3)	Sesn2 ↑ *	RT-qPCR; WB	[207]
**Respiratory Disease**					
Tracheal airway tissue	Ovalbumin-induced asthma	Asthma (*n* = 10) vs. control (*n* = 10)	Sesn2 ↑ *	RT-qPCR	[208]
Airway smooth muscle cells	Asthma	Asthma (*n* = 3) vs. control (*n* = 3)	Sesn2 ↑ *	RT-qPCR	[208]
**Degenerative**					
Spinal cord (L4-6 dorsal horn)	Osteoarthritis pain (MIA model)	MIA OA (*n* = 6) sham control (*n* = 6)	Sesn2 – (NS)	WB	[209]
**Musculoskeletal Remodelling/Atrophy**					
Skeletal muscle (plantaris)	Skeletal muscle hypertrophy (synergist ablation model)	Overload-induced hypertrophy (*n* = 9) vs. sham control (*n* = 9)	Sesn2 ↓ *	WB	[210]
Skeletal muscle (soleus)	Disuse-induced skeletal muscle atrophy	3-day hindlimb suspension (*n* = 8) vs. control (*n* = 8)	Sesn3 ↓ *	RT-qPCR	[211]
**Physiological/Experimental conditions**					
Liver	Short-term calorie restriction	Short-term calorie restriction (*n* = 4–6) vs. control (*n* = 4–6)	Sesn1 – Sesn2 – Sesn3 – (NS)	WB	[212]
Liver	Thyroid hormone (T3) treatment	24 h after T3-treatment (*n* = 3–7) vs. control (*n* = 3–7)	Sesn2 ↑ *	RT-qPCR	[213]
Skeletal muscle (quadriceps)	Ageing with lifelong exercise training	Lifelong aerobic training (*n* = 4) vs. control (*n* = 4)	Sesn1 ↑ * Sesn2 ↑ * Sesn3 ↑ *	RT-qPCR	[214]
Skeletal muscle (quadriceps)	Ageing with lifelong exercise training	Lifelong aerobic training (*n* = 3) vs. control (*n* = 3)	Sesn1 ↑ *	WB	[214]
Skeletal muscle (soleus)	Ageing with lifelong exercise training	Lifelong aerobic training (*n* = 4) vs. control (*n* = 4)	Sesn1 – Sesn2 – Sesn3 – (NS)	RT-qPCR	[214]
Skeletal muscle (soleus)	Ageing with lifelong exercise training	Lifelong aerobic training (*n* = 3) vs. control (*n* = 3)	Sesn1 – Sesn2 – Sesn3 – (NS)	WB	[214]
Skeletal muscle (gastrocnemius)	Aerobic exercise training	Aerobic exercise (*n* = 12) vs. sedentary control (*n* = 12)	Sesn2 – (NS)	WB	[215]
Skeletal muscle (vastus lateralis)	Early-life stress (maternal separation model)	Maternal separation (*n* = 18) vs. control (*n* = 18)	Sesn3 ↓ *	RT-qPCR	[216]
Skeletal muscle (vastus lateralis)	Chronic unpredictable stress	CUS (*n* = 10) vs. control (*n* = 10)	Sesn3 – (NS)	RT-qPCR	[216]
Cortical neurons	Activity-dependent neuronal stimulation	BiC and 4-AP synaptic treatment (*n* = 3) vs. control neurons (*n* = 3)	Sesn2 ↑ *	RT-qPCR	[217]
Neonatal cardiac fibroblasts	Vasoactive peptide hormone stimulation	Angiotensin II-treatment (*n* = 3) vs. control (*n* = 3)	Sesn1 ↓ *	RT-qPCR; WB	[218]

**Table 4:** Circulating Sestrins and their change in serum or plasma. Summary of published studies reporting circulating SESN1, SESN2, or SESN3 concentrations measured in human serum or plasma across different clinical conditions. “Sestrin change” indicates the direction of expression relative to the study-defined control group. Values are reported as mean ± standard deviation or median (interquartile range) as presented in the original studies. Symbols denote the reported direction of change (↑, upregulation; ↓, downregulation; –, no change). Statistical significance is presented as reported in the original studies (NS, not significant; NR, not reported). Values marked with an asterisk (*) were visually inferred from figures when numerical values were not explicitly reported in the text or tables of the original article. Abbreviations: AD, Alzheimer’s disease; AMI, acute myocardial infarction; AoD, aortic dissection; CAD, coronary artery disease; CAS, carotid atherosclerosis; CHD, coronary heart disease; COPD, chronic obstructive pulmonary disease; CTRL, control; DM, diabetes mellitus; DN, diabetic nephropathy; DPN, diabetic peripheral neuropathy; ELISA, enzyme-linked immunosorbent assay; HF, heart failure; MCI, mild cognitive impairment; OSA, obstructive sleep apnea; PAS, placenta accreta spectrum; PCOS, polycystic ovary syndrome; PD, Parkinson’s disease; RRMS, relapsing-remitting multiple sclerosis; RET, resistance training; SAP, stable angina pectoris; SE, sepsis; SICM, sepsis-induced cardiomyopathy; SPR, surface plasmon resonance; SS, septic shock; T2DM, type 2 diabetes mellitus; TPL, threatened preterm labour; UAP, unstable angina pectoris.

Condition	Specimen (Assay)	Study Group (*n*)	Sestrin Concentration (ng/mL)	Sestrin Change	Statistical Test	Ref.
**Diabetes**						
Diabetic neuropathy (T2DM complication)	Serum (ELISA)	Control (*n* = 39)	9.10 (5.41, 13.53)	–	–	[219]
		T2DM (*n* = 49)	14.58 (7.93–26.62)	SESN2 ↑	(*p* < 0.05)	
		DPN (*n* = 47)	9.86 (6.72–21.71)	SESN2 ↑	T2DM vs. DPN (*p* < 0.05)	
Diabetic nephropathy (T2DM complication)	Serum (ELISA)	Control (*n* = 30)	8.04 ± 0.76	–	–	[220]
		DM with normoalbuminuria (*n* = 30)	6.47 ± 0.86	SESN2 ↓	(*p* < 0.05)	
		DM with microalbuminuria (*n* = 30)	5.27 ± 0.61	SESN2 ↓	(*p* < 0.05)	
		DM with macroalbuminuria (*n* = 30)	4.02 ± 0.47	SESN2 ↓	(*p* < 0.05)	
Diabetic nephropathy (T2DM complication)	Serum (ELISA)	Control (*n* = 20)	5.9 ± 1.9	–	–	[221]
		DM with normoalbuminuria (*n* = 22)	5.6 ± 2.9	SESN2 ↓	ANOVA (*p* < 0.05)	
		DM with microalbuminuria (*n* = 35)	3.7 ± 1.7	SESN2 ↓	ANOVA (*p* < 0.05)	
		DM with macroalbuminuria (*n* = 19)	3.1 ± 1.4	SESN2 ↓	ANOVA (*p* < 0.05)	
Diabetic nephropathy (T2DM complication)	Serum (ELISA)	Control (*n* = 20)	2.32 ± 0.55	–	–	[222]
		DM with normoalbuminuria (*n* = 20)	1.62 ± 0.36	SESN2 ↓	(*p* < 0.05)	
		DM with microalbuminuria (*n* = 20)	1.02 ± 0.18	SESN2 ↓	(*p* < 0.05)	
		DM with macroalbuminuria (*n* = 20)	0.55 ± 0.29	SESN2 ↓	(*p* < 0.05)	
Type 2 diabetes	Plasma (ELISA)	Control (*n* = 326)	8.25 ± 7.57	–	–	[223]
		Diabetes (*n* = 518)	5.49 ± 5.94	SESN2 ↓	(*p* < 0.05)	
Type 2 diabetes and dyslipidemia	Serum (ELISA)	Control (*n* = 46)	0.7063 ± 0.077	–	–	[224]
		DM (*n* = 40)	0.375 ± 0.045	SESN2 ↓	(*p* < 0.05)	
		Dyslipidemia (*n* = 42)	0.4152 ± 0.0447	SESN2 ↓	(*p* < 0.05)	
		DM with dyslipidemia (*n* = 41)	0.3192 ± 0.0263	SESN2 ↓	(*p* < 0.05)	
Type 2 diabetes with/without carotid atherosclerosis	Serum (ELISA)	Control (*n* = 46)	5.32 (4.32, 6.79)	–	–	[225]
		DM without CAS (*n* = 114)	5.27 (4.3,6.37)	SESN2 –	NS (*p* = 0.308)	
		DM with CAS (*n* = 80)	5.66 (4.42,6.96)	SESN2 –	NS (*p* = 0.308)	
Type 2 diabetes with/without coronary heart disease	Plasma (ELISA)	T2DM without CHD (*n* = 70)	11.17 (9.79, 13.14)	–	–	[226]
		T2DM with CHD (*n* = 69)	9.46 (8.34, 10.91)	SESN2 ↓	(*p* < 0.05)	
**Metabolic**						
Paediatric obesity with/without type 2 diabetes	Serum (ELISA)	Control (*n* = 136)	5.8 ± 1.8	–	–	[227]
		Obese without DM (*n* = 90)	4.1 ± 2.6	SESN2 ↓	(*p* < 0.05)	
		Obese with T2DM (*n* = 72)	2.9 ± 1.4	SESN2 ↓	(*p* < 0.05)	
Paediatric obesity	Plasma (ELISA)	Control (*n* = 36)	0.6 (0.27–1.4)	–	–	[228]
		Obese (*n* = 34)	0.3 (0.03–0.7)	SESN2 ↓	(*p* < 0.05)	
**Cardiovascular**						
Aortic dissection	Plasma (ELISA)	Control (*n* = 40)	0.83 (0.61, 1.20)	–	–	[36]
		Stanford A (*n* = 70)	0.92 (0.69, 1.69)	SESN2 ↑	(*p* < 0.05)	
		Stanford B (*n* = 50)	1.00 (0.74, 1.32)	SESN2 ↑	(*p* < 0.05)	
Carotid atherosclerosis (carotid plaque severity)	Plasma (ELISA)	Plaque score = 0 (*n* = 89)	12.8 (11.2, 15.6)	–	Overall (*p* < 0.05)	[229]
		Plaque score = 1 (*n* = 31)	12.7 (11.4, 16.3)	SESN2 –	Score 1 vs. Score 0 (NS)	
		Plaque score ≥2 (*n* = 32)	15.9 (13.6, 20.0)	SESN2 ↑	Score ≥2 vs. Score 0 (*p* < 0.05)	
Coronary artery disease	Plasma (ELISA)	Control (*n* = 129)	14.2 (12.8, 17.9)	–	–	[230]
		Coronary artery disease (*n* = 175)	16.4 (13.0, 20.7)	SESN2 ↑	(*p* < 0.05)	
Coronary artery disease (SAP, UAP, AMI)	Plasma (ELISA)	Chest pain without CAD (*n* = 35)	16.34 ± 3.59	–	–	[231]
		SAP (*n* = 44)	22.50 ± 5.51	SESN1 ↑	(*p* < 0.05)	
		UAP (*n* = 41)	28.84 ± 10.04	SESN1 ↑	(*p* < 0.05)	
		AMI (*n* = 29)	33.24 ± 11.77	SESN1 ↑	(*p* < 0.05)	
Coronary artery disease (SAP, UAP, AMI)	Plasma (ELISA)	Chest pain without CAD (*n* = 35)	6.91 ± 0.93	–	–	[231]
		SAP (*n* = 44)	7.99 ± 1.24	SESN3 ↑	(*p* < 0.05)	
		UAP (*n* = 41)	9.20 ± 1.39	SESN3 ↑	(*p* < 0.05)	
		AMI (*n* = 29)	9.85 ± 2.07	SESN3 ↑	(*p* < 0.05)	
Left-to-right shunt congenital heart disease (with/without heart failure)	Serum (ELISA)	Control (*n* = 30)	7.06 ± 2.22	–	–	[232]
		Left-to-right shunt CHD without HF (*n* = 36)	15.09 ± 5.03	SESN2 ↑	(*p* < 0.05)	
		Left-to-right shunt CHD with HF (*n* = 16)	20.22 ± 5.18	SESN2 ↑	(*p* < 0.05)	
**Inflammatory/Infectious**						
Hashimoto’s disease (autoimmune, inflammation)	Serum (ELISA)	Control (*n* = 64)	1.83 (1.34–2.64)	–	–	[233]
		Hashimoto’s disease (*n* = 110)	1.36 (1.10–2.03)	SESN2 ↓	(*p* < 0.05)	
Kawasaki disease (paediatric systemic vasculitis)	Serum (ELISA)	Control (*n* = 38)	3.49 (2.98, 6.14)	–	–	[234]
		Kawasaki disease (*n* = 72)	5.17 (3.90, 7.91)	SESN2 ↑	(*p* < 0.05)	
Rheumatoid arthritis	Serum (ELISA)	Control (*n* = 55)	18.26 ± 7.08	–	–	[235]
		Rheumatoid arthritis (*n* = 55)	10.38 ± 4.03	SESN1 ↓	(*p* < 0.05)	
Sepsis	Serum (ELISA)	ICU patients without infection (*n* = 14)	2.8 ± 0.6	–	–	[236]
		Sepsis (*n* = 42)	5.3 ± 2.8	SESN2 ↑	(*p* < 0.05)	
Sepsis and septic shock	Serum (ELISA)	Control (*n* = 50)	15.82 ± 3.59	–	–	[237]
		SE group (*n* = 63)	10.14 ± 2.59	SESN2 ↓	(*p* < 0.05)	
		SS group (*n* = 47)	6.36 ± 1.44	SESN2 ↓	(*p* < 0.05)	
Septic shock with/without septic cardiomyopathy	Serum (ELISA)	Control (*n* = 67)	5.8 (5.1, 6.6)	–	–	[238]
		SS non-SICM (*n* = 127)	14.6 (9.1, 19.2)	SESN2 ↑	(*p* < 0.05)	
		SS SICM (*n* = 61)	9.1 (7.3, 17.6)	SESN2 ↑	(*p* < 0.05)	
**Ischemic/Injury Models**						
Acute ischemic stroke (AIS)	Plasma (ELISA)	Control (*n* = 30)	8.383 ± 7.39	–	–	[239]
		Acute ischemic stroke (*n* = 187)	1.434 ± 3.57	SESN2 ↓	(*p* < 0.05)	
Acute mountain sickness (AMS+ vs. AMS- after ascent)	Plasma (ELISA)	AMS- (*n* = 39)	4.04 ± 2.67	–	–	[240]
		AMS+ (*n* = 13)	5.27 ± 3.55	SESN2 –	NS (*p* = 0.078)	
**Ageing and Senescence**						
Age-related frailty (Fried frailty index)	Serum (ELISA)	Not frail (*n* = 79)	9.2 (3.7–11.25)	–	–	[241]
		Fried frailty phenotype (*n* = 138)	10.57 (5.20–11.91)	SESN1 ↑	(*p* < 0.05)	
Age-related frailty (Rockwood frailty index)	Serum (SPR)	Non-frail (*n* = 41)	17.61 ± 0.55 (16.5–18.7)	–	–	[242]
		Rockwood frailty (*n* = 51)	14.58 ± 0.34 (13.9–15.3)	SESN1 ↓	(*p* < 0.05)	
Age-related frailty (Rockwood frailty index)	Serum (SPR)	Non-frail (*n* = 41)	14.14 ± 0.41 (13.3–14.9)	–	–	[242]
		Rockwood frailty (*n* = 51)	12.74 ± 0.30 (12.1–13.3)	SESN2 ↓	(*p* < 0.05)	
**Neurodegeneration/Neurological disease**						
Alzheimer’s disease and mild cognitive impairment	Serum (SPR)	Control (*n* = 60)	13.82 ± 0.27	–	–	[243]
		MCI (*n* = 27)	17.17 ± 0.33	SESN2 ↑	(*p* < 0.05)	
		AD (*n* = 41)	18.36 ± 0.25	SESN2 ↑	(*p* < 0.05)	
Major depressive disorder	Serum (ELISA)	Control (*n* = 77)	2.51 ± 0.67	–	–	[244]
		Major depressive disorder (*n* = 153)	2.17 ± 0.61	SESN2 ↓	(*p* < 0.05)	
Parkinson’s disease	Serum (SPR)	Control (*n* = 54)	13.65 ± 2.125	–	–	[245]
		PD group (*n* = 36)	15.96 ± 2.428	SESN2 ↑	(*p* < 0.05)	
Relapsing remitting multiple sclerosis	Serum (ELISA)	Control (*n* = 45)	2.54 (1.36–9.52)	–	–	[246]
		RRMS (*n* = 40)	1.64 (0.91–2.47)	SESN2 ↓	(*p* < 0.05)	
**Respiratory Disease**						
Asthma (exacerbated and controlled state)	Plasma (ELISA)	Control (*n* = 32)	1.32 ± 0.48	–	–	[247]
		Exacerbated asthma (before treatment) (*n* = 44)	1.75 ± 0.53	SESN2 ↑	(*p* < 0.05)	
		Controlled asthma (after treatment) (*n* = 44)	1.56 ± 0.46	SESN2 ↑	(*p* < 0.05)	
COPD	Serum (ELISA)	COPD without emphysema (*n* = 27)	1.09 (0.9, 1.9)	–	–	[248]
		COPD with emphysema (*n* = 40)	6.7 (2.7, 10.9)	SESN2 ↑	(*p* < 0.05)	
COPD	Serum (ELISA)	Control (*n* = 62)	5.00 ± 3.93	–	–	[249]
		COPD (*n* = 62)	8.61 ± 2.89	SESN2 ↑	(*p* < 0.05)	
**Degenerative/Musculoskeletal Remodelling/Atrophy**						
Age-related sarcopenia	Serum (SPR)	Non-sarcopenics (*n* = 52)	17.55 ± 0.70	–	–	[250]
		Sarcopenics (*n* = 50)	17.37 ± 0.42	SESN1 ↓	(*p* < 0.05)	
Age-related sarcopenia	Serum (SPR)	Non-sarcopenics (*n* = 52)	13.97 ± 0.58	–	–	[250]
		Sarcopenics (*n* = 50)	7.50 ± 0.41	SESN2 ↓	(*p* < 0.05)	
Radiographic axial spondyloarthritis (r-axSpA)	Serum (ELISA)	Control (*n* = 48)	23.22 ± 18.68	–	–	[251]
		r-axSpA (*n* = 48)	15.62 ± 4.72	SESN1 ↓	(*p* < 0.05)	
**Reproductive/Maternal Health**						
Endometrial polyps and uterine leiomyomas	Serum (ELISA)	Control (*n* = 59)	1.10097	–	–	[252]
		Polyp (*n* = 60)	1.94128	SESN2 ↑	(*p* < 0.05)	
		Myoma (*n* = 57)	1.9124	SESN2 ↑	(*p* < 0.05)	
Ovarian endometrioma	Serum (ELISA)	Control (*n* = 43)	5.57 ± 1.52	–	–	[253]
		Endometrioma (*n* = 37)	9.32 ± 2.59	SESN2 ↑	(*p* < 0.05)	
Placenta accreta spectrum	Serum (ELISA)	Control (*n* = 26)	0.89 ± 0.25	–	–	[254]
		PAS group (*n* = 41)	1.0 ± 0.45	SESN2 –	NS	
Polycystic ovary syndrome	Serum (ELISA)	Control (*n* = 90)	15.18 ± 10.91	–	–	[255]
		Non-obese PCOS (*n* = 90)	8.19 ± 4.94	SESN2 ↓	(*p* < 0.05)	
		Obese PCOS group (*n* = 90)	6.42 ± 4.05	SESN2 ↓	(*p* < 0.05)	
Polycystic ovary syndrome	Serum (ELISA)	Control (*n* = 32)	255.78 (25.5–528.7)	–	–	[256]
		PCOS (*n* = 31)	40.74 (24.4–257.7)	SESN2 ↓	(*p* < 0.05)	
Polycystic ovary syndrome	Serum (ELISA)	Control (*n* = 30)	3.38 (0.4)	–	–	[257]
		PCOS (*n* = 30)	6.2 (0.8)	SESN2 ↑	(*p* < 0.05)	
Polycystic ovary syndrome	Serum (ELISA)	Control (*n* = 46)	1.348 ± 0.549	–	–	[258]
		PCOS (*n* = 37)	1.665 ± 0.671	SESN2 ↑	(*p* < 0.05)	
Preeclampsia	Serum (ELISA)	Control (*n* = 30)	0.749 (0.60)	–	–	[259]
		Preeclampsia (*n* = 26)	0.733 (0.35)	SESN2 –	NS	
		Severe preeclampsia (*n* = 24)	1.891 (4.83)	SESN2 ↑	(*p* < 0.05)	
Threatened preterm labour (TPL)	Serum (ELISA)	Healthy pregnancies (*n* = 26)	22.82 ± 3.097	–	–	[260]
		TPL with term delivery (≥37 wk) (*n* = 27)	5.90 ± 1.59	SESN2 ↓	(*p* < 0.05)	
		TPL with preterm delivery (<37 wk) (*n* = 27)	1.98 ± 0.76	SESN2 ↓	(*p* < 0.05)	
Uterine leiomyoma	Serum (ELISA)	Control (*n* = 30)	5.8 ± 1.3	–	–	[261]
		Myoma (*n* = 31)	11.7 ± 2.5	SESN2 ↑	(*p* < 0.05)	
**Physiological/Experimental conditions**						
Resistance training	Serum (ELISA)	No training, Baseline (*n* = 12)	Baseline 0.3 ± 0.17	–	Interaction NS (*p* = 0.1029)	[262]
		No training, Follow-up (*n* = 12)	Follow-up 0.34 ± 0.16	SESN2 –		
		Resistance training, Baseline (*n* = 13)	Pre-training baseline 0.39 ± 0.16	SESN2 –		
		Resistance training, Follow-up (*n* = 13)	Post-training follow-up 0.61 ± 0.24	SESN2 ↑	Δ RET vs. Δ CTRL (*p* < 0.05)	
**Sleep Disorders**						
Chronic insomnia disorder	Serum (ELISA)	Control (*n* = 56)	5.4 (4.6, 6.4)	–	–	[263]
		Chronic insomnia disorder (*n* = 65)	5.1 (4.1, 5.8)	SESN2 ↓	(*p* < 0.05)	
Obstructive sleep apnea	Serum (ELISA)	Control (*n* = 26)	~2.2 *	–	–	[264]
		OSA (*n* = 38)	~4 *	SESN2 ↑	(*p* < 0.05)	
Obstructive sleep apnea	Plasma (ELISA)	Control (*n* = 21)	2.06 ± 1.76	–	–	[265]
		Mild (*n* = 7)	2.63 ± 1.66	SESN2 –	NS	
		Moderate (*n* = 10)	3.09 ± 1.77	SESN2 ↑	(*p* < 0.05)	
		Severe (*n* = 19)	5.28 ± 2.36	SESN2 ↑	(*p* < 0.05)	

**Table 5:** Summary of Sestrin expression patterns across disease categories and biological sample types. Direction of change is indicated as increased (↑), decreased (↓), or no available data (–). Where available, trends are further qualified: ‘all ↑/all ↓’ indicates that all included studies report the same direction of change; ‘majority ↑/majority ↓’ indicates that most studies report the same trend with limited conflicting evidence; ‘overall ↑/overall ↓’ indicates mixed findings, with more studies supporting one direction than the other; and ‘mixed’ indicates no clear consensus across studies.

Condition	Human Tissue (Biopsy/Cells)	Human Serum/Plasma	Mouse Tissue (Biopsy/Cells)	Rat Tissue (Biopsy/Cells)
Diabetes	mixed, overall ↓ with complication	majority ↓ (lower with severity)	majority ↓	majority ↓
Metabolic	mixed, overall ↓ (disease-specific)	all ↓	mixed (stage-specific)	all ↓
Cardiovascular	mixed (disease-specific)	all ↑ (higher with severity)	all ↑	mixed
Inflammatory/infectious	mixed (acute ↑, chronic ↓)	mixed	majority ↑	mixed (stage-specific)
Ageing/senescence	mixed (tissue ↓, immune cells ↑)	mixed, overall ↓	majority ↓	mixed (tissue-specific)
Ischemia/injury	–	overall ↓	mixed, overall ↑ (stage-specific)	mixed (tissue- and model-specific)
Neurodegenerative/ neurological	majority ↑	mixed (disease-specific)	mixed (model-dependent)	all ↑
Degenerative	majority ↓	all ↓	all ↑	no difference (single study)
Musculoskeletal remodelling/atrophy	↓ (single study)	–	mixed	all ↓
Exercise	all ↑	↑ (single study)	all ↑	mixed (tissue-specific)
Respiratory	overall ↑	all ↑ (higher with severity)	–	all ↑
Environmental/external stressors	majority ↑ (acute ↑, chronic ↓)	—	mixed (model-specific)	—
Reproductive	—	mixed (disease-specific)	—	—
Sleep	—	overall ↑	—	—

## Data Availability

No new data were created or analysed in this study.

## Supplementary Materials

The following supporting information can be downloaded at https://www.mdpi.com/article/10.3390/cells15070651/s1. Table S1: “Extended—Circulating Sestrins and their change in serum or plasma”.
